# An epigenome-wide association study of early-onset major depression in monozygotic twins

**DOI:** 10.1038/s41398-020-00984-2

**Published:** 2020-08-25

**Authors:** Roxann Roberson-Nay, Dana M. Lapato, Aaron R. Wolen, Eva E. Lancaster, Bradley T. Webb, Bradley Verhulst, John M. Hettema, Timothy P. York

**Affiliations:** 1 Department of Psychiatry, Virginia Commonwealth University, Richmond, VA USA; 2 Virginia Commonwealth University, Virginia Institute for Psychiatric and Behavioral Genetics, Richmond, VA USA; 3 Department of Human and Molecular Genetics, Virginia Commonwealth University, Richmond, VA USA; 4 Department of Surgery, Transplant Research Institute, University of Tennessee Health Science Center, Memphis, TN USA; 5 Department of Psychology, Michigan State University, East Lansing, MI USA

**Keywords:** Comparative genomics, Genomics, Depression

## Abstract

Major depression (MD) is a debilitating mental health condition with peak prevalence occurring early in life. Genome-wide examination of DNA methylation (DNAm) offers an attractive complement to studies of allelic risk given it can reflect the combined influence of genes and environment. The current study used monozygotic twins to identify differentially and variably methylated regions of the genome that distinguish twins with and without a lifetime history of early-onset MD. The sample included 150 Caucasian monozygotic twins between the ages of 15 and 20 (73% female; *M*age = 17.52 *SD* = 1.28) who were assessed during a developmental stage characterized by relatively distinct neurophysiological changes. All twins were generally healthy and currently free of medications with psychotropic effects. DNAm was measured in peripheral blood cells using the Infinium Human BeadChip 450 K Array. MD associations with early-onset MD were detected at 760 differentially and variably methylated probes/regions that mapped to 428 genes. Genes and genomic regions involved neural circuitry formation, projection, functioning, and plasticity. Gene enrichment analyses implicated genes related to neuron structures and neurodevelopmental processes including cell–cell adhesion genes (e.g., *PCDHA genes*). Genes previously implicated in mood and psychiatric disorders as well as chronic stress (e.g., *NRG3*) also were identified. DNAm regions associated with early-onset MD were found to overlap genetic loci identified in the latest Psychiatric Genomics Consortium meta-analysis of depression. Understanding the time course of epigenetic influences during emerging adulthood may clarify developmental phases where changes in the DNA methylome may modulate individual differences in MD risk.

## Introduction

Major depression (MD) is highly prevalent, ranking second in the global burden of disease, with the overall lifetime risk estimated to be 16.2% in the general population^1^. MD is associated with increased mortality, particularly suicide^1^. Among adolescents, MD is associated with the greatest level of impairment of all psychiatric conditions, with 16% of females and 12% of males endorsing at least one major depressive episode (MDE) by age 18^2^. An early age of onset confers increased risk for negative socioemotional outcomes including recurrent MDEs^3,4^. Adolescence/young adulthood is characterized by neurophysiological changes (e.g., synaptic pruning, myelination) that significantly influence brain function and behavior, which may increase risk for MD and other psychiatric conditions^5^. Thus, understanding the genetic contributions to MD during this dynamic neurophysiological period where peak incidence is observed^2,6–8^ is critical to elucidating developmentally informed pathways to mood disorders.

Twin and family studies robustly demonstrate that genetic factors play a role in risk for MD, with heritability estimates of roughly 35% for MD and 45% for early-onset MD^9^; these heritability estimates indicate that MD is suitable for epigenetic study using twins^10^. Moreover, a large number of genetic loci have been identified for MD, supporting the role of genetic factors in the etiology of MD^11,12^. Twin study variance component analyses also indicate a considerable contribution of unique environmental risk factors to MD^9^. Due to the substantial link between environmental hardship and onset of an MDE^13,14^, epigenetic mechanisms may, in part, mediate the influence of environmental stress and combine with genetic liability to increase MD risk over the lifespan^15–17^. Epigenetic mechanisms refer to DNA, chromatin, and RNA modifications that can influence the expression of genes but do not alter the underlying genetic sequence. Animal studies have been critical to establishing a causal association between early life environments, epigenetic alterations, and phenotypic outcomes. For example, the seminal work of Michael Meaney and his research team reveals the importance of maternal care in altering the expression of genes that regulate behavioral and neuroendocrine responses to stress as well as synaptic development in the rat hippocampus^18–23^. Indeed, a number of animal and human studies demonstrate lasting epigenetic alterations occurring in the genomes of cells including changes to postmitotic neurons that integrate experience-dependent changes^24^. Thus, the timing of environmental stress plays an important role in subsequent epigenetic consequences, with early life stress paradigms in mice and humans demonstrating enduring changes in epigenetic profiles^18,21,25–29^.

A number of studies have utilized genome-wide platforms to determine DNA methylation (DNAm) differences between MD cases and controls. However, as much as 37% of DNAm variance can be accounted for by genetic factors^30^ with recent studies indicating that common genetic variation (i.e., methylation quantitative trait loci [*mQTLs*]) influence DNAm levels^31–35^. Most MD case-control studies of DNAm do not account for allelic variation, which means genetic and environmental influences on DNAm cannot be disaggreagated. In contrast, the quasi-experimental design afforded by monozygotic (MZ) twins greatly improves on the unmatched case-control design (see Supplementary Table 1). The use of MZ twins, both discordant and concordant pairs for outcome, adjusts for much of the impact of unmeasured confounds such as genetic variation, uterine environment, age, sex, race, cohort effects, and exposure to many shared environmental events.

The current study utilized the robust MZ twin design coupled with an analytically powerful approach to detect differentially and variably methylated DNAm regions associated with early-onset MD in a sample of adolescent and emerging adult twins. Studying this developmental period offers a number of advantages over later life periods, including fewer confounds to DNAm variability such as a history of prolonged or multiple psychiatric/medical comorbidities and medication usage as well as long-term nicotine use. Moreover, it eliminates the well-known epigenetic changes associated with aging^36–38^. The developmental window of young adulthood also is associated with moderate conservation of DNAm that is nonetheless responsive to environmental signals^39^, making it an ideal sensitive period for the study of MD.

## Methods

### Participants

One hundred sixty-six MZ twins (83 pairs) were drawn from a larger sample of twins (*N* = 430 pairs) enrolled in a parent study (R01MH101518)^40^, which was designed to estimate genetic and environmental contributions to RDoC Negative Valence Systems constructs using classical twin modeling (i.e., disaggregating variance into additive genetic, common shared, and unique non-shared environmental variation)^41–51^. Blood samples were collected as part of the parent R01 for future use. Twins were primarily recruited through the Mid-Atlantic Twin Registry (MATR), a population-based registry^40,41^. All participants (parents/minor children, adults) provided written informed consent/assent.

Of the 83 twin pairs initially identified as MZ via the zygosity questionnaire, five pairs (6.3%) were determined to be DZ pairs using DNA-based markers and were removed (for zygosity determination, see Supplementary). One or both twins from three additional pairs failed DNA-based quality control checks, reducing the final analyzed sample to 75 MZ twin pairs (150 twins; see Table 1 for details). Of the 150 twins, 39 twins endorsed a history of at least one MD episode, resulting in 27 discordant, 42 concordant negative (i.e., both twins MD unaffected), and 6 concordant positive (i.e., both twins MD affected) pairs. All twins were raised together in the same home and were required to be free of psychotropic medications/medications with psychotropic effects at the time of study entry, although ~4.0% (*n* = 6) endorsed a history of psychotropic medication use, slightly lower than the national average^42^. See full study exclusionary criteria in Supplementary material.

**Table 1:** Demographic and clinical characteristics of twins meeting definite or probable DSM-5 criteria for lifetime history of MD (MD Affected) versus no lifetime history of MD (MD Unaffected).

	*M* (SD) or *n* (%)			
	MD unaffected *n* = 111	MD affected *n* = 39	*t/χ*^*2*^	*P*
Demographic/Sample				
Age, years	17.49 (1.3)	17.60 (1.3)	0.66	0.51
Sex, female	81 (73.0%)	29 (74.4%)	0.28	0.87
thnicity, Hispanic	5 (4.5%)	3 (7.7%)	0.58	0.43
Nicotine use^a^, current smoker	2 (1.8%)	3 (7.7%)	3.11	0.11
Clinical characteristics				
SMFQ	4.4 (3.7)	9.0 (6.0)	6.40	<0.001
History of psychotropic medication use	2 (1.8%)	4 (10.3%)	0.64	0.62
Panic attack^b^	10 (9.0%)	6 (15.4%)	1.19	0.28
Social anxiety disorder	10 (9.1%)	8 (20.5%)	3.54	0.08
Specific phobia	8 (7.3%)	7 (17.9%)	3.63	0.07
Generalized anxiety disorder	2 (1.8%)	4 (10.3%)	5.31	0.04
MD features				
Age of onset (years)	–	14.9 (1.7)		
Number of major depressive episodes, *n*				
1 episode	–	17 (43.6%)		
2–3 episodes	–	16 (41.0%)		
4–5 episodes	–	3 (7.7%)		
≥6 episodes	–	3 (7.7%)		
Time since last MDE (years)	–	1.31 (1.7)		
Number of symptoms during worst MDE	–	5.69 (1.1)		

*SMFQ* Short Mood and Feelings Questionnaire.

^a^For smokers, Fagerstrom test for nicotine dependence scores ranged 2 (low dependence) to 6 (moderate dependence) with a Mode = 2, Median = 2, and Mean = 3.

^b^No case meet full criteria for panic disorder.

### Measures

#### Major depression

All twin pairs completed a psychiatric history based on an expanded version of the Composite International Diagnostic Interview—Short Form, which queried DSM-5 MD Criterion A and C (see Supplementary for questions and diagnostic algorithm)^43^. Current depressive symptoms also were assessed using the Short Mood and Feelings Questionnaire (SMFQ), which is a 13-item questionnaire validated to measure depressive symptoms in adolescents and adults^44^.

### DNAm measurements

Genomic DNA was isolated from peripheral blood according to standard methods using the Puregene DNA Isolation Kit (Qiagen). An aliquot of 1 µg DNA per subject was processed for bisulfite conversion (Zymo Research EZ Methylation Kit) and genome-wide DNAm assayed on the Infinium Human Methylation 450 K BeadChip microarray, which interrogates 485,512 features. Twin pairs were localized to the same slide to minimize any potential artefactual differences in DNAm patterns due to batch effects.

Details of the 450 K microarray have been previously described^45^, and raw data processing was performed according to best practices reported in recent publications^46,47^. Intensity values from the scanned arrays were processed using the *minfi* Bioconductor package^48^ in the R programming environment (R Development Core Team 2015). There were no sample outliers in values of ancestry-based principal components estimated from ancestry informative DNAm probes^49^.

Quality was assessed both quantitatively and visually to identify samples with poor signal intensity^48^. Beta values were derived as the ratio of the methylated probe intensity to the sum of the methylated and unmethylated probe intensities^50^. Beta value density plots from each array were inspected to tag poor performing arrays based on a large deviation from the rest of the samples. Probes were filtered if they had a detection *P* value of greater than 0.01 in at least 10% of samples or if they have been previously identified as cross-hybridizing^51^, leaving a total of 455,828 probes to analyze (Supplementary Fig. 1). Quantile normalization adapted to DNAm arrays^52^ was applied to adjust the distribution of type I and II probes to the final set of screened sample arrays and probes.

For all statistical tests, beta values were transformed using the *M*-value procedure to promote normality and calculated as a logit transformation of the methylated and unmethylated intensity ratio along with an added constant to offset potentially small values^50^. Correlations between major experimental factors and the top ten principal components of *M*-values across all arrays were inspected to identify extraneous structure that may account for any batch effects^53^. ComBat was used to adjust for differences across arrays due to slide groupings^54^. Blood cell type proportions were inferred for each sample to account for cellular heterogeneity using the Houseman method^55^.

### Analytic approach

The current sample was composed of three types of MZ twin pairs (i.e., discordant MD, concordant positive, concordant negative), resulting in a number of potential statistical contrasts to be tested (see Supplementary Table 1). Rather than test each concordance group separately, which substantially reduces statistical power, we used all data in one comprehensive linear mixed-effects model, which generates results similar to a paired *t*-test^56^. All contrasts were simultaneously fit within one linear model, allowing the filtering out of CpGs (i.e., cytosine nucleotide linked to guanidine nucleotide by phosphate) emerging from inconsequential contrasts not of interest (i.e., contrasts 2–5). For example, contrasts 4 and 5 compare CpGs within twins of a concordant negative pair and a concordant positive pair, respectively. This approach retains all the advantages of a typical discordant MZ twin design with the added benefit of including negative controls by inclusion of the set of concordant pairs. This conservative approach was taken by retaining only those probes that were unique to the primary contrast of interest (MD affected versus MD unaffected).

Within the linear model, lifetime early-onset MD status and natural killer (NK) cell proportion served as fixed effects, and a random effect term was included to account for the correlated structure of twin pair membership. NK cell proportion was the only estimated cell type nominally significantly different between MD cases and controls (*t* = 0.266, *p* = 0.051) and, therefore, it was included as a covariate to control for potential bias that might arise from DNAm differences due to changes in NK blood cell proportions rather than those attributable to DNAm changes associated with MD itself. Sex and age were not included as fixed effects given no association with MD (age, *p* = 0.53; sex, *p* = 0.87).

### Identifying regional DNAm change

Based on observations that the average correlation between probes on the 450 K microarray within ~250 base pairs (bp) is 0.83 and within 1 kb is 0.45^57–59^, several methods have been proposed to take advantage of this structure to identify consistent DNAm change across a contiguous region^57,58,60,61^. Current approaches quantify regional DNAm change either as a mean difference (differentially methylated region [DMR]) or as a difference in variance (variably methylated region [VMR]). Due to the sparse and highly clustered placement of features on the Illumina 450 K platform, a custom approach for defining DMRs and VMRs was developed for this study adapted from the algorithm proposed by Ong et al.^46,57^. They suggest both a regional and individual CpG probe approach run in tandem since ~25% of probes do not have a neighboring probe within 1 kb. To this end, the single-probe analysis provided the raw materials for the regional approach adopted to identify and assess significance of DMRs and VMRs. Type I error rates were estimated from empirical *P* values for all univariate statisitics (mean level and variance based tests) described below were calculated using a permutation approach. For *k* = 1000 reordering’s, the outcome variable was resampled in a way that preserved the discordance/concordance pair status frequencies. The false discovery rate (FDR)^62^ was estimated from the distribution of these empirical *P* values.

#### Differentially methylated regions

Univariate tests were performed by fitting a linear mixed-effects model^63^ separately for each probe by regressing the normalized DNAm probe intensity on lifetime MD status while adjusting NK cell proportion. A random effect term was included to account for the correlated structure of twin pair membership. Probes were filtered if they had a *P* value < 0.05 in any contrast not of interest. From this reduced set, differentially methylated probes (DMPs) were called significant at an FDR < 0.01 in the contrast of interest (Supplementary Fig. 1). DMRs were constructed using the same univariate test statistics. Regions were built with univariate tests filtered for statistics in the 1st and 99th quantile for effects not of interest and retained for statistics in the 5th and 95th quantile for the effect of interest. (Supplementary Fig. 2) From this set of CpG probes, a candidate DMR was defined as having at least 2 contiguous CpGs within 1 kb. The strength of a DMR association was estimated by a test statistic reflecting the area of influence approximated by the trapezoidal rule where the height (*h*) was the length in base pairs between two contiguous CpGs and *a* and *b* were the univariate test statistic for the contiguous CpGs. For DMRs with more than two CpGs, the area for each contiguous pairing was summed to represent the area under the curve (AUC) for the entire DMR. All probes in the DMR had the restriction of test statistics with the same sign. A positive test statistic indicated hypermethylation in cases versus controls while a negative test statistic indicated hypomethylation.

#### Variably methylated regions

A similar strategy was adopted to identify VMR (see Supplementary Fig. 2). In this case, the univariate test statistic calculated was the *F*-value comparing the variance of two samples, cases versus controls. Probes were filtered if they had a *P* value < 0.05 in any contrast not of interest. From this reduced set, VMPs were called significant at an FDR < 0.01 in the contrast of interest. VMRs were constructed using the same univariate test statistics. Regions were built with univariate tests filtered for statistics in the 1st and 99th quantile for effects not of interest and retained for statistics in the 10th and 90th quantile for the effect of interest. VMRs were restricted to at least two contiguous probes within 1 kb whereby all probes had the restriction of either increasing or decreasing variability in cases versus controls. The strength of regional influence for variance differences was estimated using the trapezoidal rule, as was used previously for DMRs.

#### Significance assessment of regional change

The statistical significance of DMR and VMR AUC estimates were assessed using a rank-based permutation method^64^. This nonparametric method estimates an FDR without relying on strong assumptions about the normality of the data. The *k* = 1000 permutations of univariate tests were used to estimate the expected order statistics. The FDR was calculated based on the observed versus expected null scores. Briefly, for a range of thresholds, regions are called significant if the value of the observed ordered test statistic minus the mean value from the permuted rank exceeded a given threshold. The number of falsely called regions is the median number of regions that exceed the lowest AUC value of regions called significant. The FDR is calculated as the ratio of the number of falsely called regions to the number of regions called significant. An implementation of the SAM algorithm is available as an R package^65^ but was recoded to allow for flexibility in specifying models for the twin data and to trim extreme test statistics likely to be false positives before the calculation of the FDR.

### Functional and regulatory enrichment

The distribution of significant CpG probes and regions identified to be differentially and variably methylated by MD status were examined separately across functional and regulatory annotations. CpG findings were mapped to known genes^66^ for enrichment of Gene Ontology classifications^67^ using clusterProfiler^68^. Classification functions included biological processes, cellular components, and molecular function, in addition to KEGG pathways. Tests for nonrandom association of CpG island features and ChromHMM chromatin states were based on the AH5086 and AH46969 tracks from the AnnotationHub package^69^, respectively. CpG island shores were defined as being 2 kb regions flanking CpG islands while shelves were demarcated as 2 kb upstream or downstream shore regions^70^. A test of enrichment for each of these annotations was calculated by comparing the proportion of sequence from the intersection of significant CpG regions with the regions defined by the annotation feature. Bootstrap methods using 1000 resamplings were used to estimate 95% confidence intervals. This observed overlap was compared to an empirical distribution of random samples of genome groups of the same size and structure drawn from the background set under consideration. Empirical *P* values were calculated from 1000 random reorderings of the data using standard methods^71^.

### PGC GWAS enrichment

A similar resampling method was performed to count the number of significant CpG regions that overlapped with the findings of a recent genome-wide association study (GWAS) meta-analysis conducted by the Psychiatric Genomics Consortium (PGC) group^11^. Specifically, data from the Wray et al. study^11^ which specified the coordinates for the linkage disequilibrium (LD) blocks of each of the 44 hits was used. The region boundaries of the DMPs/DMRs/VMPs/VMRs to the allelic LD block boundaries was compared. Only direct overlaps were included (i.e., being close to the boundary was not sufficient to count as an overlap). The depression phenotype in this meta-analysis is derived from a number of different methods including clinical interview, self-report, electronic medical record abstraction, and self-report of a lifetime diagnosis. This study identifies 44 MD-associated loci across 18 chromosomes, which includes genes enriched for targets of antidepressant medication. The nonrandom frequency of overlap between the significant CpG regions and the 44 independent PGC findings was assessed using bootstrap and permutation approaches from 1000 data resamplings.

### Blood–brain DNAm associations

The majority of epigenetic studies conducted to date have relied on peripheral blood as the primary tissue source, but use of this tissue has been questioned in the context of psychiatric disorders where the primary tissue of interest is brain. Use of peripheral tissues is necessary and knowing whether DNAm markers in the periphery mirror those in the brain is key. Thus, the Image-CpG database^72^ was used to determine which peripheral blood loci were informative markers of the brain for measurements of DNAm. The Image-CpG database includes DNAm data derived from four tissues including brain, blood, saliva, and buccal cells from a sample of medically intractable epilepsy patients. Resected brain tissue samples were acquired from multiple brain regions including the temporal cortex, hippocampus, amygdala, frontal cortex, frontal lobe, and insular cortex; sampled brain regions varied by participant. Correlations are available for DNAm measurements using the Infinium HumanMethylation450 chip. Summary statistics are provided to the public including Spearman rho correlations and *p* values for tissue pairs. For the purpose of this paper, we were interested in the blood–brain tissue correlations for all significant CpGs located in DMRs and VMRs. Thus, the median rho for CpGs included in each DMR and VMR was computed as well as the minimum and maximum rho for CpGs.

## Results

### Sample characteristics

MZ twins meeting DSM-5 MD criteria at the probable or definite level self-reported higher depression symptom scores on the SMFQ and had higher rates of generalized anxiety disorder compared to MD unaffected twins (*t*(1,146) = 6.4, *p* < 0.001); *χ*^2^(1) = 5.31, *p* = 0.04, respectively; Table 1). For those twins meeting DSM-5 criteria for at least one MDE, the mean age at onset was ~15 years, and the majority of twins (~85%) reported experiencing 3 or fewer MDEs in their lifetime. Most MD affected twins (82%) reported five or more symptoms during their worst lifetime MDE. The mean length of time since last MDE was 1.31 years (SD = 1.7, range: 0–6.67 years), with 63% of MD affected twins experiencing their last MDE in the past year. Participant age, sex, self-reported ethnicity, and smoking behavior did not differ by MD status. See the Supplementary for additional analyses related to smoking.

### Differentially methylated probes and regions

From the set of 455,828 screened CpG probes, 50,990 background regions could be created, covering 59.9 megabases. Following univariate tests, a total of 3995 regions consisting of 28,600 CpG probes could be considered candidate DMRs (see Supplementary Fig. 1. From these, seventeen DMRs were identified as significantly associated with MD (all hypermethylated in MD cases) of which 15 mapped onto genes (FDR < 10%; see Table 2). The number of CpG probes in significant DMRs ranged from 3 to 7 (median = 4). Individual probe testing (DMP) resulted in 59 hypomethylated and 77 hypermethylated CpG sites with respect to MD status (FDR < 1%; Supplementary Table 2). The combined set of 30.6 kb DNA sequence covered by significant DMR and DMP findings had a nonrandom pattern of enrichment across ChromHMM annotations, specifically sites of strong transcription (*p* = 0.019), enhancers (*p* = 0.045), ZNF genes/repeats (*p* = 0.001), heterochromatin (*p* = 0.013), and weak repressed polycomb (*p* = 0.045) (Supplementary Fig. 3) and CpG island relationships which included both north (*p* = 0.024) and south (*p* = 0.003) shelf regions (Supplementary Fig. 4). Effect sizes (*R*^2^) were computed for all DMPs, with a median *R*^2^ of 0.063 (range 0.024–0.139; see Supplementary Table 2 for DMP *R*^2^ values).

**Table 2:** Differentially methylated regions (DMRs) where MD affected twins exhibited higher means compared to MD unaffected twins and blood–brain correlations^a^ for CpGs within a DMR.

Chr	Start	End	Symbol	EntrezID	Number CpGs	Empirical AUC	Median Bl–Br Corr	Min Bl–Br Corr	Max Bl–Br Corr
chr1	36787678	36789401	*SH3D21*	79729	3	3836.37	−0.21	−0.44	−0.18
chr1	221053841	221055665	*HLX*	3142	4	4242.35	0.01	−0.18	0.61
chr2	240035107	240036791	*HDAC4*	9759	3	4057.64	−0.08	−0.16	0.15
chr5	1090741	1092417	*SLC12A7*	10723	4	3818.33	0.08	−0.09	0.43
chr5	171709917	171711524	*UBTD2*	92181	3	4070.73	0.43	0.04	0.55
chr5	176936563	176938522	*DOK3*	79930	6	3826.30	−0.33	−0.47	0.09
chr6	30519905	30521619	NA	NA	5	3861.90	0.41	−0.19	0.55
chr6	31828260	31830030	NA	NA	4	4558.54	0.40	0.03	0.54
chr6	32797253	32798887	NA	NA	4	3849.67	0.24	−0.18	0.44
chr6	39281541	39283313	*KCNK17*	89822	3	4122.08	−0.03	−0.04	0.26
chr6	146863647	146865487	*RAB32*	10981	7	4701.74	−0.22	−0.53	0.27
chr16	2023998	2025868	*TBL3*	10607	4	4329.89	0.20	−0.02	0.44
chr16	58767249	58769104	*GOT2*	2806	4	4046.93	0.02	−0.58	0.24
chr17	46659019	46660940	*HOXB3*	3213	4	4501.78	−0.05	−0.24	0.03
chr17	70116185	70118162	*SOX9*	6662	4	4120.22	0.27	−0.10	0.50
chr19	13213428	13215387	*LYL1*	4066	3	4381.78	−0.33	−0.44	0.04
chr21	38069321	38070994	NA	NA	4	3892.48	−0.18	−0.45	0.02

*Bl* blood, *Br* brain.

^a^Correlations were obtained from the ImageCpG databaseAM^72^.

### Variably methylated probes and regions

Regional analysis identified ten VMRs significant from a total of 11,055 candidate regions (FDR < 17%). Seven of the VMRs mapped onto genes (Table 3). Significant VMRs were all more variable in MD cases, and the number of CpG probes in these regions ranged from 2 to 11 (median = 4). The VMP analysis yielded 560 significant VMP findings (FDR < 1%), all of which were more variable in MD cases except for a single probe (Supplementary Table 3). The combined set of VMR and VMP DNA regions of 16.6 Kb reflected a nonrandom enrichment with ChromHMM annotations for 5′/3′ transcription (*p* = 0.035), genic enhancers (*p* = 0.024), and heterochromatin (*p* = 0.050) (Supplementary Fig. 5) and CpG island relationships within the south shelf (*p* = 0.029) (Supplementary Fig. 6).

**Table 3:** Variably methylated regions (VMRs) where MD affected twins exhibited greater variance compared to MD unaffected twins and blood–brain correlations^a^ for CpGs within a VMR.

Chr	Start	End	Symbol	EntrezID	Number CpGs	Empirical AUC	Median Bl–Br Corr	Min Bl–Br Corr	Max Bl–Br Corr
chr3	39321449	39323539	*CX3CR1*^b^	1524	4	5572.92	0.67	−0.10	0.82
chr3	46925081	46925524	*PTH1R*	5745	2	5031.13	0.04	−0.12	0.19
chr3	170136920	170137321	*CLDN11*	5010	2	5110.24	−0.12	−0.45	0.22
chr6	32049516	32049825	NA	NA	3	5038.87	0.18	0.18	0.27
chr6	169284344	169287304	NA	NA	5	5276.01	0.27	−0.04	0.31
chr6	170595385	170597898	*DLL1*	28514	8	5628.62	0.38	0.16	0.61
chr7	157225062	157225567	NA	NA	2	5131.48	0.81	0.79	0.83
chr10	134739746	134741032	*CFAP46*	54777	4	5667.46	0.13	−0.30	0.16
chr11	64510112	64513156	*RASGRP2*	10235	7	5458.44	−0.12	−0.37	0.84
chr17	40837037	40839469	*CNTNAP1*	8506	4	5035.57	0.35	0.16	0.48

*Bl* blood, *Br* brain.

^a^Correlations were obtained from the ImageCpG database^72^.

^b^Missing one CpG from ImageCpG database.

### Gene enrichment analysis

Genes that mapped to significant differentially or variably methylated findings were combined for gene-based enrichment to provide an overview of all DNAm contributions at a functional level. The results of enrichment tests yielded significant over-representation for biological processes (BP) and cellular function (CF), and no enrichment for molecular function or KEGG pathways, at FDR < 10% (Table 4). The BP gene category associations were hemophilic cell adhesion and cell–cell adhesion while the significant terms for CF were associated with functions of neurons, including neuron projection terminus, terminal button, axon part, cell projection part, axon, and presynapse.

**Table 4:** Gene enrichment analysis summary.

	Ontology category	Description	Gene ratio	*q*-value	Gene symbol
GO:0007156	Biological process	Homophilic cell adhesion via plasma membrane adhesion molecule	23, 568	0.00002	*CDH3, CDH6, PCDHAC1-2, PCDHA1-13, PCDH10, PCDH20, PTPRT, CADM1, PLXNB3, CLSTN2*
GO:0098742	Biological process	Cell–cell adhesion via plasma membrane adhesion molecules	28, 568	0.00002	*CDH3, CDH6, PTPRT, CLDN4, CADM1, GRID2, ITGA5, CLDN11, PLXNB3, PCDHAC1-2, PCDHA1-13, PCDH20, PTPRD, CLSTN2*
GO:0044306	Cellular function	Neuron projection terminus	14, 605	0.05888	*BAIAP2, AAK1, CYFIP1, SYT11, PACSIN1, ILK, PFN2, PNOC, PVALB, DNAJC5, MOB2, NAPA, BSN, AP3D1*
GO:0043195	Cellular function	Terminal bouton	9, 605	0.05888	*AAK1, CYFIP1, SYT11, ILK, PFN2, PVALB, DNAJC5, NAPA, AP3D1*
GO:0033267	Cellular function	Axon part	16, 605	0.07314	*AAK1, TRAK1, CYFIP1, SYT11, KIF13B, AP3M1, NRG1, ILK, PFN2, PVALB, SPG7, DAGLA, DNAJC5, CNTNAP1, NAPA, AP3D1*
GO:0044463	Cellular function	Cell projection part	54, 605	0.08617	*AKAP9, RASGRP2, BAIAP2, DCTN2, IGF2BP1, EHD1, CNGA4, PACRG, EPS8, ENKUR, SPATA13, AAK1, TRAK1, CYFIP1, SYT11, KIF13B, TBC1D30, GPR161, AP3M1, NPHP3, BBS9, GRID2, PACSIN1, NRG1, ILK, ITGA5, KCNC3, PDE6B, PFN2, PNOC, SSH1, CFAP46, CCDC40, PRKAR1B, TTYH1, PTH1R, TENM2, PVALB, BBS2, MAP2K4, SLC22A5, SPG7, TIMP2, DAGLA, CA9, JADE1, DNAJC5, MOB2, CNTNAP1, NAPA, BSN, AP3D1, DHRS3, RAB28*
GO:0030424	Cellular function	Axon	26, 605	0.08617	*IGF2BP1, DAB2IP, EPHB2, AAK1, TRAK1, CYFIP1, SYT11, KIF13B, LDLRAP1, AP3M1, NRG1, ILK, KCNC3, NF1, PFN2, SLC17A7, SEMA6A, PVALB, MAP2K4, SPG7, DAGLA, DNAJC5, CNTNAP1, NAPA, BSN, AP3D1*
GO:0098793	Cellular function	Presynapse	24, 605	0.08617	*BAIAP2, AAK1, CYFIP1, SYT11, DMXL2, AMPH, GRIK4, GRM8, PACSIN1, HIP1, APBA2, ILK, KCNC3, NF1, SYT17, PFN2, PNOC, PI4K2A, SLC17A7, PVALB, DNAJC5, NAPA, BSN, AP3D1*

### Relationship between early-onset MD DNAm markers and PGC MD-associated genetic Loci

**Fig. 1:**
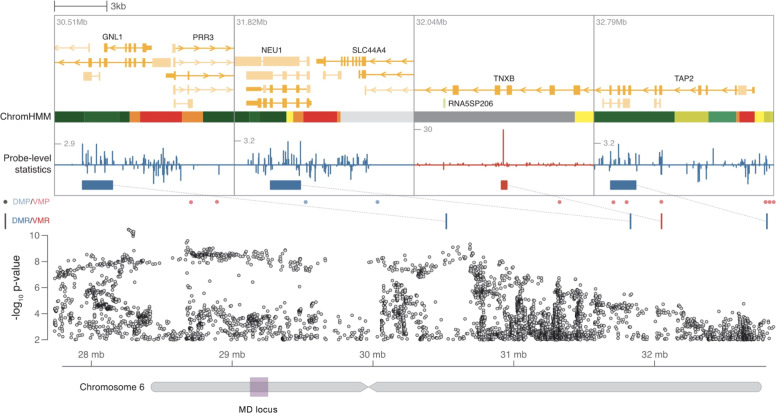
Overlap with PGC GWAS for major depression. The ‘MD’ locus (purple box) represents a region of chromosome 6 extending from 27.7 to 32.8 Mb found to be significantly associated with depression by Wray et al. Summary statistics from this study are plotted for the relevant regional markers in the Manhatten plot. Colored ticks represent the 3 DMPs (blue) and 1 VMR (red) located in this region. Individual plots above provide a zoomed-in view of the genomic context surrounding each methylation region and probe level test statistic. Chromatin states within GM12878 lymphoblastoid cells are indicated by color coding the ChromHMM track.

A total of 6 differentially methylated sites (including DMP/DMRs; *p* = 0.002; 95% CI = 2–11) (Supplementary Table 4) and 12 variably methylated sites (including VMPs/VMRs; 95% CI = 5–19, *p* = 0.008) (Supplementary Table 4) overlapped significant PGC GWAS loci. These enrichment results were largely driven by overlap observed with the PGC GWAS locus on chromosome 6 at 27.738–32.848 Mb (Fig. 1). At this locus, 5 of 6 differentially and 10 of 12 variably methylated sites overlapped.

### Relationship between blood and brain DNAm markers

Tables 2 and 3 include the median Spearman rho correlation as well as minimum and maximum correlation for CpGs included within each DMR and VMR, respectively. Across all DMRs, the median correlation between blood and brain CpGs ranged −0.33 to 0.43. The magnitude of the minimum and maximum correlations was similar (rho = −0.58 and rho = 0.61, respectively). For VMRs, the median correlation between blood and brain CpGs ranged −0.12 to 0.67. The minimum rho observed was −0.45 while the maximum rho was 0.84.

## Discussion

The main objective of the current study was to identify differentially and variably methylated positions and regions that distinguished MZ twins with and without a history of early-onset MD. Across BP and CF domains of the gene enrichment analysis, there was consistency in the functional attributes of the genes related to neural structures and processes as a key differentiating feature between MD affected and unaffected MZ twins. BP gene ontologies referenced homophilic cell adhesion and cell–cell adhesion processes, which are involved in brain functioning.

A number of cadherins and protocadherins emerged in the cell adhesion gene sets with MD affected persons demonstrating increased variation in two cadherins (*CDH3*, *CDH6*), the clustered protocadherin alpha family (*PCDHA*), and two non-clustered protocadherins (*PCDH10, PCDH20*). CDHs/PCDHs are a group of calcium dependent cell–cell adhesion molecules that are abundantly expressed in the nervous system and play a major role in multiple steps essential to neurodevelopment (e.g., dendrite arborization, synaptogenesis^73–78^). The DNAm profile of the *PCDH*s is known to be responsive to environmental factors^79^, and emerging evidence suggests a role for *PCDH*s in multiple psychiatric phenotypes including MD^80,81^. Two small twin studies analyzing peripheral blood samples report an association between several cadherin/protocadherin genes and twin pairs repeatedly discordant for elevated depression symptoms^82^ as well as a history of MD or an anxiety disorder^83^. A related study observed increased DNAm in *PCDH* gene families with the highest enrichment of hypermethylated sites in the *PCDHA* genes located in the hippocampus of suicide completers with a history of severe childhood abuse^20^. At the genetic variant level, a recent meta-analysis detected an SNP (rs9540720) in the non-clustered *PCDH9* gene to be significantly associated with MD case status at the genome-wide level^84^, and a related gene that encodes a protein of the same family (*PCDH17)* was found to confer risk for mood disorders^85^. Moreover, expression patterns of *Pcdh* genes have been examined in rodent brain regions involved in the neurocircuitry of MD^86^, with results indicating high expression levels in subregions of the hippocampus and basolateral amygdaloid complex^86,87^. The clustered *PCDHA* family also is strongly expressed in serotonergic neurons^88–90^ and *PcdhαC2* is necessary for axonal tiling and assembly of serotonergic circuitries^90^. Thus, a comprehensive understanding of the genetic architecture of the developing adolescent/young adult brain may be critical to identify etiological determinants of MD.

Additional genes/gene regions previously linked to MD, stress, or psychiatric/substance use disorders differentiated twins with and without a lifetime history of MD (e.g., *HDAC4, NRG3, CRHR2*)^91–97^. Moreover, 6 differentially methylated and 12 variably methylated findings overlapped loci identified in a recent PGC GWAS of depression. These enrichment results were primarily driven by regions in the extended MHC region chromosome 6, which has been associated with both depression, bipolar disorder, and schizophrenia^98,99^. The MHC also is densely populated with genes related to neuronal signaling and plays a role in immunity. Other genes related to immune functioning (e.g., *CX3CR1*, *IL23R*)^100–103^ also emerged. Study results support multiple etiologic pathways to MD and indicate that complex genetic disorders such as MD likely reflect a large number of independent genetic factors that each contribute a small amount of variance to disease susceptibility and multiple psychiatric diseases likely share genetic risk factors.

The ImageCpG database was used to determine which peripheral blood loci were revealing of brain for measurements of DNAm. While unique and informative, the Image-CpG dataset includes brain and blood correlations from a small sample of ten subjects with medically intractable epilepsy. Moreover, while tissue was gathered from several of the most optimal brain regions involved in the pathophysiology of MD (e.g., amygdala, hippocampus), there was varation in brain tissue collection across participants and not all brain regions relevant to MD were assessed (e.g., nucleus accumbens). Nonetheless, this valuable dataset provided an opportunity to determine the correspondence between blood and brain DNA markers. Strong correlations were observed for a number CpGs, indicating a reasonable level of resemblance between brain and blood. The most striking blood–brain association was observed for the VMR of the *CX3CR1* gene (median rho = 0.67 and maximum rho = 0.82). The magnitude of association also was generally higher for CpGs involved in VMRs compared to DMRs and some correlations were robustly negative, indicating blood–brain relationships where CpGs are hypermethylated in one tissue and hypomethalated in the other. Overall, a number of markers exhibited promising associations, supporting the potential of blood to track MD.

Strengths of the current study design include its use of both discordant and concordant MZ twins in conjunction with a sensitive developmental window to reveal genome-wide DNAm biomarkers associated with early-onset MD. Limitations of the current study include its reliance on an all Caucasian sample, which may reduce the generalizability of findings. Consistent with epidemiological findings^104^, females represented the majority of the sample. Thus, increased numbers of males are needed to determine potential sex-related differences. The cross-sectional design of the current study also does not allow for determination of epigenetic differences as cause or consequence of MD onset. Finally, like many DNAm studies, DNA was derived from a peripheral tissue (i.e., blood) versus brain tissue, with the latter being the tissue of primary interest for psychiatric disorders. Nonetheless, these results add to the literature, showing an association between the early life major depression and differential DNAm patterns. As a next step, it will be necessary to characterize the longitudinal course of epigenetic influences during emerging adulthood to clarify how the DNA methylome contributes to the pathoetiology of MD.

## Web resources

Image-CpG database: http://han-lab.org/methylation/default/imageCpG.

## Supplementary information

Supplement

## Data Availability

Code will be made available to interested parties upon request.
