# Supplementary material for: An epigenome-wide association study of early-onset major depression in monozygotic twins
Source: Transl Psychiatry. 2020 Aug 25;10:301. doi: 10.1038/s41398-020-00984-2 (PMC7447798; doi:10.1038/s41398-020-00984-2)
Supplement: Supplementary file 1 — Supplement [file 41398_2020_984_MOESM1_ESM.docx]

**SUPPLEMENTAL**

**METHODS**

The primary definition of MD affected status required presence of at least one DSM-5 MDE lasting at least two weeks^1^. MD was considered present if criteria were met at either a definite or a probable level. A definite diagnosis was made when a twin endorsed at least five DSM-5 Criterion A MDE symptoms (e.g., low energy, recurrent thoughts of death, inability to concentrate) of which at least one symptom had to include the presence of low mood and/or anhedonia most of the day, nearly every day for at least two weeks during their worst episode. Probable diagnoses were made when a twin endorsed at least 4 symptoms, one of which had to include the presence of low mood and/or anhedonia most of the day, nearly every day. The use of definite and probable levels allows for the inclusion of twins who may not be currently symptomatic and, therefore, have to rely on retrospective memory to determine their symptom presentation. The use of probable levels of diagnosis also is justified given that twins expressing nearly all symptoms of a MDE are more similar to a case than a control symptomatically and for depressive risk factors^2^. This approach to diagnosis was introduced as part of the Research Diagnostic Criteria and has been applied in many studies^3–6^. To qualify for MD at the definite or probable level, participants also had to report that their depression symptoms caused clinically significant distress or functional impairment (i.e., Criterion C). MD unaffected status was defined as no lifetime diagnosis of MD at the probable or definite threshold. The expanded MD section of the CIDI-SF followed by the lifetime MD algorithm is presented below.

**Lifetime Major Depression Diagnostic Questions:**

**Criterion A (MDE Symptoms)**

1. Have you ever had a time in your life when you felt sad, blue, or depressed or two weeks or more in a row? (yes/no)
2. Have you ever had a time in your life lasting two weeks or more when you lost interest in most things like hobbies, work, or activities that usually give you pleasure? (yes/no)
3. During those worst two weeks, did the feelings of sadness or loss of interest usually last all day long, most of the day, about half of the day, or less than half of the day? (All day long, Most of the day, About half of the day, Less than half of the day)
4. Did you feel this way every day, almost every day, or less often during the two weeks? (Every day, Almost every day, Less often)

If a participant responded “yes” to questions 1 OR 2 AND endorsed “all day long” or “most of the day” to question 3 AND responded “every day” or “almost every day” to question 4, they entered the MD section and were queried regarding other DSM-5 Criterion A symptoms of MD (see below).

1. Thinking about those same two weeks, did you feel more tired out or low on energy than is usual for you? (yes/no)
2. Did you gain or lose weight without trying or did you stay about the same? (Gained weight, Lost weight, Gained and lost weight, stayed about the same, I was on a diet)
3. About how much weight did you gain/you lose/your weight change? (numeric response)
4. Did you have more trouble falling asleep or staying asleep than you usually do during those two weeks? (yes/no)
   1. Did that happen every night, nearly every night, or less often during those two weeks? (Every night, Nearly every night, Less often)
5. During those two weeks, did you have a lot more trouble concentrating or making decisions than usual? (yes/no)
6. People sometimes feel down on themselves, no good, or worthless, or have excessive guilt and blame themselves for things. During that two-week period, did you feel this way? (yes/no)
7. Did you think a lot about death – either your own, someone else’s, or death in general during those two weeks? (yes/no)

**Criterion C (Functional Interference):**

1. Did you ever tell a professional about these problems (such as a medical doctor, psychologist, social worker, counselor, nurse, clergy, or other helping professional)? (yes/no)
2. Did you ever take medication for these problems? (yes/no)
3. How much did these problems interfere with your life or activities – a lot, some, a little, or not at all? (A lot, Some, A little, Not at all)

*additional questions regarding age of onset, timing of last episode, etc. were queried.

**MD Algorithm**

Questions 6 and 7 were recoded so that a response of “Gained weight” or “Gained and lost weight,” along with at least a response of 5 pounds in question 7, was coded as endorsement of weight gain/loss. Participants who endorsed question 8 also were presented with question 8a. Sleep symptoms were considered present if a participant answered “Every night” or “Nearly every night” on question 8a. Responses to queries 1, 2, 5, weight gain, sleep symptoms, 9, 10, and 11 were summed as the total number of depression symptoms endorsed during the twin’s worst MDE. Participants were deemed to have experienced significant distress if they endorsed question 14 as “a lot” or “some” and/or if they endorsed question 12 or 13 as “yes”. MD at the full threshold level was coded positive if the participant endorsed question 1 or 2 along with question 3 as “All day long” or “Most of the day,” and question 4 as “Every day” or “Almost every day,” and at least 4 other depression symptoms for a total of at least 5 symptoms. MD at the full threshold level also required significant distress. MD was coded positive at the probable level if the participant endorsed question 1 or 2 as well as question 3 (“All day long” / “Most of the day”) and question 4 (“Every day” / “Almost every day”) and at least 3 other depression symptoms for a total of 4 symptoms. The probable level also required endorsement of significant distress (i.e., endorsed question 12, 13, or 14 as “a lot” or “some”).

**Exclusionary Criteria**

Participants were not eligible for the current study if they met any of the following criteria: 1) current use of psychotropic medications (e.g., antianxiety/antidepressants) or medications with psychotropic effects (e.g., beta-adrenergic blockers), b) diagnosis of an autism spectrum disorder, c) diagnosis of an intellectual disability, d) diagnosis of a spatial learning disorder, or prior testing indicating an IQ below 70, e) seizure without a clear and resolved etiology, f) current or past episodes of psychosis, g) serious, not stabilized illness (e.g., liver, kidney, gastrointestinal, respiratory, cardiovascular, endocrinologic, neurologic, immunologic, or blood disease), h) inadequate production of human growth hormone, i) sensory integration disorder, j) congenital adrenal hyperplasia, k) adrenal inefficiency, l) deaf with bicochlear implants, m) cancer (current or past diagnosis), and n) pregnancy (current or lifetime). If only one twin from the twin pair met any of the exclusionary criteria, the whole pair was excluded.

**Zygosity**

Zygosity status (monozygotic [MZ] versus dizygotic [DZ]) for adolescent twins (age < 17) was determined based on parent-report about physical similarities between twins. Adult twins (age > 18) not accompanied by a parent/legal guardian completed the zygosity questionnaire about themselves. Prior research has demonstrated high validity for this zygosity assessment as compared to blood^7^ and DNA evaluations of zygosity^8^. MZ zygosity based on questionnaire assessment was confirmed using 65 SNP control probes included on the Infinium HumanMethylation450 (450K) array to verify sample identity. The control probes target polymorphic sequences, and values for each probe cluster into three groups corresponding to genotype. Together, the control probes provide strong support for validating zygosity. Only data from twins confirmed to be MZ were included in analyses.

**Smoking**

### In addition to the MD affected versus MD unaffected group comparison for rate of smoking, the current sample’s methylation data was examined to determine the potential influence of smoking on DNAm patterns. No single probe or any element of the enrichment analyses suggests that smoking influenced this sample’s DNAm data. Specifically, the CG05951221 (AHRR gene) probe, which is most robustly associated with smoking, yielded a q-value of 0.98 (p-value = 0.65). Moreover, the enrichment results are not similar to the gene ontology categories (e.g., pulmonary traits, cancer pathways, cardiovascular disease) cited in well-powered DNAm studies of smoking^9^.

Supplemental Figure 1. Flowchart describing work flow of DMP/DMR generation.


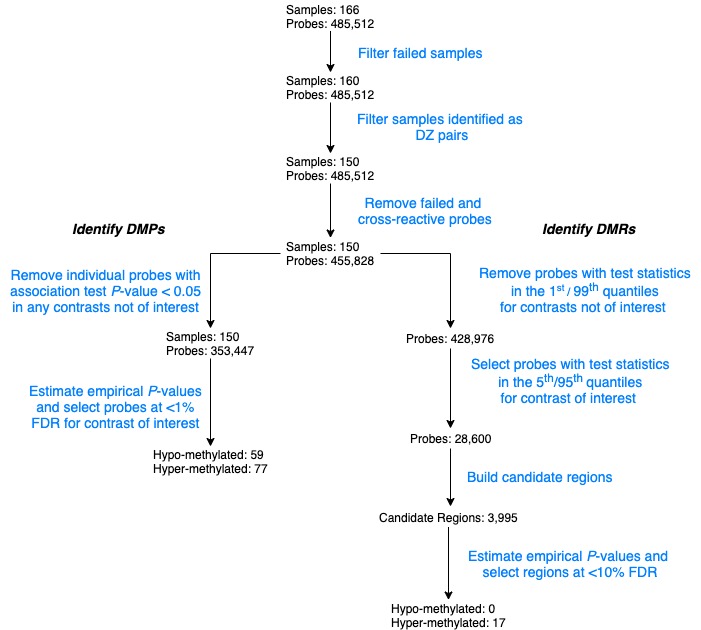


Supplemental Figure 2. Flowchart describing work flow of VMP/VMR generation.

**
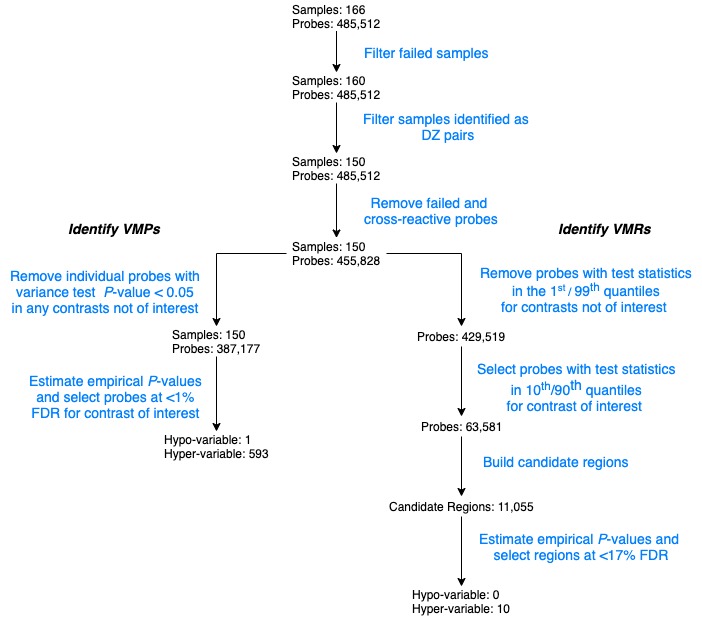
**

**Supplemental Table 1. Statistical contrasts fit to estimate the degrees of association between MD affected/unaffected status and genome-wide DNA methylation markers.**

|  | **contrast 1** | **contrast 2** | **contrast 3** | **contrast 4** | **contrast 5** |
| --- | --- | --- | --- | --- | --- |
| **Concordant Positive T1** | -1 | -1 | 0 | -1 | 0 |
| **Concordant Positive T2** | -1 | -1 | 0 | 1 | 0 |
| **Discordant T1** | -1 | 2 | 0 | 0 | 0 |
| **Discordant T2** | 1 | 0 | 2 | 0 | 0 |
| **Concordant Negative T1** | 1 | 0 | -1 | 0 | -1 |
| **Concordant Negative T2** | 1 | 0 | -1 | 0 | 1 |

Note: T1 = Twin 1 and T2 = Twin 2; Contrast 1 is the contrast of primary interest. CpGs emerging from contrasts 2-5 were removed.

Supplemental Figure 3.

Supplemental Figure 4.

Supplemental Figure 5.

Supplemental Figure 6.

**Supplemental Table 2. DMP significant gene/promoter hits identified in the MD affected versus MD unaffected contrast.**

| **CpG** | **Chr** | **Position** | **Symbol** | **EntrezID** | **Model Coeff.** | **Unadj. Empirical P-value** | ***R^2^*** |
| --- | --- | --- | --- | --- | --- | --- | --- |
| cg22698604 | chr10 | 116286502 | *ABLIM1* | 3983 | -0.411 | 0 | 0.040 |
| cg11857921 | chr4 | 88059376 | *AFF1* | 4299 | 0.473 | 0 | 0.065 |
| cg19066150 | chr17 | 76183448 | *AFMID* | 125061 | -0.459 | 0 | 0.096 |
| cg14083554 | chr9 | 139571632 | *AGPAT2* | 10555 | 0.301 | 0 | 0.058 |
| cg02383130 | chr7 | 38670957 | *AMPH* | 273 | 0.995 | 0 | 0.097 |
| cg21980364 | chr13 | 111880998 | *ARHGEF7* | 8874 | -0.792 | 0 | 0.061 |
| cg19919209 | chr11 | 64804476 | *ARL2-SNX15* | 100528018 | 0.365 | 0 | 0.094 |
| cg20005616 | chr14 | 96829184 | *ATG2B* | 55102 | -0.424 | 0 | 0.093 |
| cg00464773 | chr17 | 79041990 | *BAIAP2* | 10458 | 0.332 | 0 | 0.086 |
| cg09347306 | chr17 | 79078315 | *BAIAP2* | 10458 | -0.333 | 0 | 0.084 |
| cg21777986 | chr18 | 60986586 | *BCL2* | 596 | 0.791 | 0 | 0.029 |
| cg00266592 | chr6 | 3139627 | *BPHL* | 670 | 0.990 | 0 | 0.048 |
| cg12597169 | chr2 | 223166094 | *CCDC140* | 151278 | -0.287 | 0 | 0.048 |
| cg07848042 | chr5 | 137667509 | *CDC25C* | 995 | -0.306 | 0 | 0.068 |
| cg20379239 | chr10 | 11207015 | *CELF2* | 10659 | -0.652 | 0 | 0.071 |
| cg11384014 | chr3 | 139658735 | *CLSTN2* | 64084 | -0.391 | 0 | 0.042 |
| cg18530956 | chr13 | 100544747 | *CLYBL* | 171425 | 0.700 | 0 | 0.064 |
| cg09159022 | chr8 | 4849522 | *CSMD1* | 64478 | 0.455 | 0 | 0.067 |
| cg22509516 | chr5 | 122848253 | *CSNK1G3* | 1456 | 0.932 | 0 | 0.058 |
| cg00029397 | chr12 | 57940957 | *DCTN2* | 10540 | -0.242 | 0 | 0.051 |
| cg27378835 | chr2 | 172950626 | *DLX1* | 1745 | 0.363 | 0 | 0.075 |
| cg12483984 | chr20 | 62545440 | *DNAJC5* | 80331 | 0.370 | 0 | 0.068 |
| cg03228065 | chr16 | 2276770 | *E4F1* | 1877 | 0.436 | 0 | 0.057 |
| cg19118812 | chr7 | 37488438 | *ELMO1* | 9844 | -0.644 | 0 | 0.057 |
| cg06833078 | chr1 | 19577645 | *EMC1* | 23065 | -0.459 | 0 | 0.093 |
| cg27054144 | chr4 | 185092121 | *ENPP6* | 133121 | -0.518 | 0 | 0.069 |
| cg11479843 | chr12 | 15941373 | *EPS8* | 2059 | 0.649 | 0 | 0.056 |
| cg16527057 | chr1 | 157069242 | *ETV3L* | 440695 | 0.353 | 0 | 0.076 |
| cg17140307 | chr1 | 93251203 | *EVI5* | 7813 | -0.736 | 0 | 0.037 |
| cg22871668 | chr6 | 133562492 | *EYA4* | 2070 | 1.053 | 0 | 0.079 |
| cg03952543 | chr6 | 123101437 | *FABP7* | 2173 | -0.349 | 0 | 0.024 |
| cg06498400 | chr10 | 15255429 | *FAM171A1* | 221061 | 0.438 | 0 | 0.079 |
| cg02356002 | chr7 | 5521483 | *FBXL18* | 80028 | 0.315 | 0 | 0.057 |
| cg25952997 | chr17 | 43299929 | *FMNL1* | 752 | -0.667 | 0 | 0.091 |
| cg05515099 | chr3 | 69482656 | *FRMD4B* | 23150 | 0.568 | 0 | 0.028 |
| cg15109851 | chr3 | 120169821 | *FSTL1* | 11167 | 1.004 | 0 | 0.088 |
| cg19934111 | chr3 | 16168679 | *GALNT15* | 117248 | -0.472 | 0 | 0.056 |
| cg08784238 | chr15 | 28511044 | *HERC2* | 8924 | -0.475 | 0 | 0.139 |
| cg18147080 | chr1 | 19244121 | *IFFO2* | 126917 | -0.567 | 0 | 0.086 |
| cg01885832 | chr17 | 47091038 | *IGF2BP1* | 10642 | 0.327 | 0 | 0.073 |
| cg07519049 | chr6 | 154830998 | *IPCEF1* | 26034 | 0.423 | 0 | 0.055 |
| cg12623544 | chr5 | 12795183 | *LINC01194* | 404663 | 0.382 | 0 | 0.047 |
| cg18951537 | chr17 | 11950516 | *MAP2K4* | 6416 | 0.424 | 0 | 0.066 |
| cg00177923 | chr8 | 29952655 | *MIR548O2* | 100616190 | -0.387 | 0 | 0.059 |
| cg01587049 | chr14 | 101492406 | *MIR758* | 768212 | -0.486 | 0 | 0.039 |
| cg07295669 | chr2 | 192275384 | *MYO1B* | 4430 | 0.361 | 0 | 0.053 |
| cg03712843 | chr13 | 39565534 | NA | NA | 0.526 | 0 | 0.102 |
| cg14685990 | chr17 | 37910900 | NA | NA | -0.318 | 0 | 0.096 |
| cg10139476 | chr8 | 39950015 | NA | NA | 0.425 | 0 | 0.093 |
| cg08701937 | chr10 | 81965488 | NA | NA | -0.593 | 0 | 0.091 |
| cg27647559 | chr11 | 58420680 | NA | NA | 0.416 | 0 | 0.085 |
| cg18379455 | chr17 | 41446167 | NA | NA | -0.338 | 0 | 0.084 |
| cg08047802 | chr12 | 108523160 | NA | NA | 0.674 | 0 | 0.08 |
| cg03808158 | chr12 | 104850326 | NA | NA | -0.772 | 0 | 0.078 |
| cg23706187 | chr4 | 127360896 | NA | NA | 0.354 | 0 | 0.077 |
| cg07203320 | chr3 | 139108984 | NA | NA | -0.382 | 0 | 0.076 |
| cg04028549 | chr16 | 85625582 | NA | NA | -0.436 | 0 | 0.073 |
| cg13294846 | chr5 | 2112351 | NA | NA | 0.569 | 0 | 0.071 |
| cg21568661 | chr15 | 62411575 | NA | NA | 0.694 | 0 | 0.071 |
| cg04507187 | chr16 | 19179258 | NA | NA | 0.490 | 0 | 0.07 |
| cg16514167 | chr11 | 12307924 | NA | NA | 0.541 | 0 | 0.07 |
| cg05522885 | chr19 | 4471596 | NA | NA | -0.634 | 0 | 0.069 |
| cg16003301 | chr12 | 56521951 | NA | NA | 1.005 | 0 | 0.067 |
| cg25248415 | chr15 | 89950204 | NA | NA | 0.573 | 0 | 0.067 |
| cg19388373 | chr2 | 11606477 | NA | NA | -0.763 | 0 | 0.066 |
| cg13826873 | chr3 | 167098267 | NA | NA | 0.652 | 0 | 0.065 |
| cg25437410 | chr5 | 178017578 | NA | NA | 1.052 | 0 | 0.064 |
| cg24476722 | chr2 | 85111399 | NA | NA | 0.388 | 0 | 0.064 |
| cg08614242 | chr10 | 103044950 | NA | NA | -0.448 | 0 | 0.062 |
| cg19159853 | chr2 | 139660149 | NA | NA | 0.375 | 0 | 0.062 |
| cg17788349 | chr2 | 131044845 | NA | NA | 0.342 | 0 | 0.06 |
| cg06040998 | chr3 | 128151940 | NA | NA | 0.560 | 0 | 0.06 |
| cg06004371 | chr7 | 33169033 | NA | NA | -0.341 | 0 | 0.06 |
| cg18542599 | chr15 | 92396808 | NA | NA | 0.681 | 0 | 0.059 |
| cg23292160 | chr4 | 154710425 | NA | NA | 0.264 | 0 | 0.057 |
| cg24538029 | chr5 | 170846083 | NA | NA | -0.419 | 0 | 0.056 |
| cg09205920 | chr6 | 29521506 | NA | NA | 0.489 | 0 | 0.055 |
| cg14323876 | chr13 | 75814627 | NA | NA | -0.606 | 0 | 0.053 |
| cg13293295 | chr2 | 69513119 | NA | NA | 0.701 | 0 | 0.051 |
| cg26445794 | chr16 | 10837274 | NA | NA | -0.622 | 0 | 0.05 |
| cg23642270 | chr4 | 11631423 | NA | NA | 0.466 | 0 | 0.048 |
| cg01832605 | chr3 | 53229991 | NA | NA | -0.278 | 0 | 0.047 |
| cg25606201 | chr5 | 180614858 | NA | NA | 0.978 | 0 | 0.047 |
| cg15047610 | chr19 | 47750787 | NA | NA | -0.610 | 0 | 0.046 |
| cg11843735 | chr16 | 68678092 | NA | NA | -0.755 | 0 | 0.046 |
| cg14186349 | chr15 | 96964022 | NA | NA | -0.680 | 0 | 0.046 |
| cg01356829 | chr1 | 67772896 | NA | NA | 0.526 | 0 | 0.04 |
| cg03763796 | chr22 | 22599128 | NA | NA | 0.435 | 0 | 0.039 |
| cg20292653 | chr2 | 5847374 | NA | NA | -0.648 | 0 | 0.037 |
| cg24449091 | chr1 | 167773027 | NA | NA | 0.363 | 0 | 0.033 |
| cg18105842 | chr17 | 7341440 | NA | NA | 0.469 | 0 | 0.03 |
| cg01426713 | chr6 | 106517533 | NA | NA | 0.701 | 0 | 0.026 |
| cg11088682 | chr3 | 172376821 | *NCEH1* | 57552 | 0.292 | 0 | 0.056 |
| cg03189678 | chr4 | 1988314 | *NELFA* | 7469 | 0.427 | 0 | 0.073 |
| cg19959519 | chr17 | 29422806 | *NF1* | 4763 | 0.381 | 0 | 0.059 |
| cg04643444 | chr1 | 31653173 | *NKAIN1* | 79570 | -0.371 | 0 | 0.057 |
| cg24015850 | chr3 | 132440674 | *NPHP3* | 27031 | -0.449 | 0 | 0.089 |
| cg05802704 | chr10 | 84175459 | *NRG3* | 10718 | 0.524 | 0 | 0.107 |
| cg15697822 | chr1 | 107684751 | *NTNG1* | 22854 | -0.715 | 0 | 0.072 |
| cg07523442 | chr6 | 163475324 | *PACRG* | 135138 | 0.418 | 0 | 0.06 |
| cg26100986 | chr7 | 100202882 | *PCOLCE* | 5118 | 0.549 | 0 | 0.044 |
| cg07886914 | chr8 | 28198639 | *PNOC* | 5368 | -0.643 | 0 | 0.034 |
| cg06238333 | chr3 | 43132062 | *POMGNT2* | 84892 | 0.462 | 0 | 0.049 |
| cg05165862 | chr12 | 50030609 | *PRPF40B* | 25766 | -0.454 | 0 | 0.068 |
| cg20073313 | chr8 | 52322009 | *PXDNL* | 137902 | -0.673 | 0 | 0.053 |
| cg09962377 | chr16 | 522007 | *RAB11FIP3* | 9727 | -0.398 | 0 | 0.07 |
| cg04113075 | chr6 | 146865487 | *RAB32* | 10981 | 0.785 | 0 | 0.039 |
| cg05367960 | chr1 | 220445206 | *RAB3GAP2* | 25782 | -0.481 | 0 | 0.044 |
| cg07781445 | chr17 | 2886250 | *RAP1GAP2* | 23108 | 0.339 | 0 | 0.031 |
| cg06035830 | chr3 | 152881217 | *RAP2B* | 5912 | -0.340 | 0 | 0.076 |
| cg05556038 | chr3 | 25427224 | *RARB* | 5915 | -0.410 | 0 | 0.082 |
| cg20721135 | chr13 | 114861488 | *RASA3* | 22821 | 0.417 | 0 | 0.042 |
| cg10413513 | chr16 | 7106265 | *RBFOX1* | 54715 | 0.380 | 0 | 0.066 |
| cg14916213 | chr11 | 66405818 | *RBM14-RBM4* | 100526737 | 0.326 | 0 | 0.046 |
| cg16670554 | chr15 | 68118985 | *SKOR1* | 390598 | 0.639 | 0 | 0.04 |
| cg13868466 | chr1 | 169455138 | *SLC19A2* | 10560 | -1.108 | 0 | 0.11 |
| cg00364615 | chr15 | 67424723 | *SMAD3* | 4088 | 0.265 | 0 | 0.034 |
| cg07135540 | chr5 | 176056414 | *SNCB* | 6620 | -0.421 | 0 | 0.064 |
| cg10876767 | chr4 | 7637533 | *SORCS2* | 57537 | 0.715 | 0 | 0.034 |
| cg18326021 | chr10 | 106401479 | *SORCS3* | 22986 | 0.687 | 0 | 0.065 |
| cg04053638 | chr2 | 84686387 | *SUCLG1* | 8802 | -0.534 | 0 | 0.05 |
| cg04017052 | chr1 | 16694728 | *SZRD1* | 26099 | -0.406 | 0 | 0.045 |
| cg14011845 | chr17 | 59533635 | *TBX4* | 9496 | -0.427 | 0 | 0.07 |
| cg09998229 | chr19 | 3606757 | *TBXA2R* | 6915 | 0.411 | 0 | 0.093 |
| cg23981116 | chr5 | 167088724 | *TENM2* | 57451 | 0.454 | 0 | 0.085 |
| cg25997756 | chr13 | 114198319 | *TMCO3* | 55002 | 0.593 | 0 | 0.069 |
| cg16770400 | chr3 | 100276538 | *TMEM45A* | 55076 | -0.403 | 0 | 0.061 |
| cg12353589 | chr11 | 1309892 | *TOLLIP* | 54472 | -0.758 | 0 | 0.076 |
| cg00287012 | chr18 | 21593174 | *TTC39C* | 125488 | 0.550 | 0 | 0.043 |
| cg02659030 | chr2 | 179396826 | *TTN* | 7273 | 0.433 | 0 | 0.05 |
| cg14373760 | chr13 | 99961037 | *UBAC2* | 337867 | -0.997 | 0 | 0.09 |
| cg25402706 | chr5 | 171641702 | *UBTD2* | 92181 | 0.833 | 0 | 0.076 |
| cg21229536 | chr5 | 82373428 | *XRCC4* | 7518 | -0.315 | 0 | 0.062 |
| cg13821577 | chr2 | 216979737 | *XRCC5* | 7520 | -0.653 | 0 | 0.082 |
| cg03202064 | chr16 | 73091917 | *ZFHX3* | 463 | 0.798 | 0 | 0.064 |
| cg04754776 | chr6 | 30031455 | *ZNRD1* | 30834 | 0.545 | 0 | 0.083 |

**Supplemental Table 3. VMP significant gene/promoter hits identified in the MD**

**affected versus MD unaffected contrast.**

| **entrez** | **symbol** | **vmp.gene** | **vmp.prom** | |  | |  | |  |
| --- | --- | --- | --- | --- | --- | --- | --- | --- | --- |
| 100128264 | *HTR5A-AS1* | 1 | NA | |  | |  | |  |
| 100128568 | *LOC100128568* | 1 | | NA | |  | |  | |
| 100131213 | *ZNF503-AS2* | 1 | 1 | |  | |  | |  |
| 100132406 | *NBPF10* | 1 | NA | |  | |  | |  |
| 100132677 | *BSN-DT* | 1 | 1 | |  | |  | |  |
| 100133612 | *LINC01134* | 1 | NA | |  | |  | |  |
| 100189589 | *DCTN1-AS1* | 1 | NA | |  | |  | |  |
| 100271715 | *ARHGEF33* | 1 | NA | |  | |  | |  |
| 100288142 | *NBPF20* | 1 | NA | |  | |  | |  |
| 100302197 | *MIR1306* | NA | 1 | |  | |  | |  |
| 100302224 | *MIR2110* | NA | 1 | |  | |  | |  |
| 100316904 | *SAP25* | 1 | NA | |  | |  | |  |
| 1004 | *CDH6* | 1 | NA | |  | |  | |  |
| 100500860 | *MIR3618* | NA | 1 | |  | |  | |  |
| 100526835 | *FPGT-TNNI3K* | 1 | NA | |  | |  | |  |
| 100529261 | *CHURC1-FNTB* | 1 | NA | |  | |  | |  |
| 100532732 | *MSH5-SAPCD1* | NA | 1 | |  | |  | |  |
| 100533179 | *UBE2F-SCLY* | NA | 1 | |  | |  | |  |
| 100533184 | *ARHGAP19-SLIT1* | 1 | NA | |  | |  | |  |
| 100534589 | *HOXA10-HOXA9* | 1 | NA | |  | |  | |  |
| 100616250 | *MIR3960* | 1 | 1 | |  | |  | |  |
| 10142 | *AKAP9* | NA | 1 | |  | |  | |  |
| 10172 | *ZNF256* | NA | 1 | |  | |  | |  |
| 10194 | *TSHZ1* | NA | 1 | |  | |  | |  |
| 10195 | *ALG3* | NA | 1 | |  | |  | |  |
| 102 | *ADAM10* | 1 | NA | |  | |  | |  |
| 10201 | *NME6* | 1 | NA | |  | |  | |  |
| 10207 | *PATJ* | 1 | NA | |  | |  | |  |
| 10217 | *CTDSPL* | 1 | NA | |  | |  | |  |
| 1025 | *CDK9* | NA | 1 | |  | |  | |  |
| 10351 | *ABCA8* | 1 | NA | |  | |  | |  |
| 10480 | *EIF3M* | 1 | 1 | |  | |  | |  |
| 10499 | *NCOA2* | 1 | NA | |  | |  | |  |
| 10500 | *SEMA6C* | NA | 1 | |  | |  | |  |
| 10516 | *FBLN5* | 1 | 1 | |  | |  | |  |
| 10522 | *DEAF1* | 1 | NA | |  | |  | |  |
| 10557 | *RPP38* | 1 | NA | |  | |  | |  |
| 10667 | *FARS2* | 1 | NA | |  | |  | |  |
| 10693 | *CCT6B* | 1 | NA | |  | |  | |  |
| 10938 | *EHD1* | 1 | 1 | |  | |  | |  |
| 11100 | *HNRNPUL1* | 1 | 1 | |  | |  | |  |
| 11122 | *PTPRT* | 1 | NA | |  | |  | |  |
| 11129 | *CLASRP* | 1 | NA | |  | |  | |  |
| 11176 | *BAZ2A* | 1 | 1 | |  | |  | |  |
| 11211 | *FZD10* | NA | 1 | |  | |  | |  |
| 11277 | *TREX1* | 1 | 1 | |  | |  | |  |
| 113091 | *PTH2* | 1 | NA | |  | |  | |  |
| 113791 | *PIK3IP1* | 1 | 1 | |  | |  | |  |
| 1141 | *CHRNB2* | 1 | NA | |  | |  | |  |
| 114815 | *SORCS1* | 1 | NA | |  | |  | |  |
| 114818 | *KLHL29* | 1 | 1 | |  | |  | |  |
| 114827 | *FHAD1* | 1 | NA | |  | |  | |  |
| 115817 | *DHRS1* | NA | 1 | |  | |  | |  |
| 116729 | *PPP1R27* | 1 | NA | |  | |  | |  |
| 116987 | *AGAP1* | 1 | NA | |  | |  | |  |
| 118424 | *UBE2J2* | 1 | NA | |  | |  | |  |
| 1186 | *CLCN7* | 1 | NA | |  | |  | |  |
| 1213 | *CLTC* | NA | 1 | |  | |  | |  |
| 124093 | *CCDC78* | NA | 1 | |  | |  | |  |
| 124401 | *ANKS3* | 1 | NA | |  | |  | |  |
| 124637 | *CYB5D1* | 1 | NA | |  | |  | |  |
| 1262 | *CNGA4* | NA | 1 | |  | |  | |  |
| 126374 | *WTIP* | NA | 1 | |  | |  | |  |
| 126868 | *MAB21L3* | NA | 1 | |  | |  | |  |
| 1284 | *COL4A2* | 1 | NA | |  | |  | |  |
| 129293 | *TRABD2A* | NA | 1 | |  | |  | |  |
| 129787 | *TMEM18* | 1 | 1 | |  | |  | |  |
| 131566 | *DCBLD2* | 1 | 1 | |  | |  | |  |
| 131965 | *METTL6* | 1 | 1 | |  | |  | |  |
| 132 | *ADK* | NA | 1 | |  | |  | |  |
| 132851 | *SPATA4* | 1 | 1 | |  | |  | |  |
| 133015 | *PACRGL* | 1 | NA | |  | |  | |  |
| 134121 | *C5orf49* | 1 | NA | |  | |  | |  |
| 134145 | *FAM173B* | NA | 1 | |  | |  | |  |
| 1364 | *CLDN4* | NA | 1 | |  | |  | |  |
| 1369 | *CPN1* | 1 | NA | |  | |  | |  |
| 1395 | *CRHR2* | 1 | NA | |  | |  | |  |
| 140739 | *UBE2F* | NA | 1 | |  | |  | |  |
| 140828 | *LINC00261* | 1 | NA | |  | |  | |  |
| 144481 | *SOCS2-AS1* | NA | 1 | |  | |  | |  |
| 1446 | *CSN1S1* | 1 | 1 | |  | |  | |  |
| 144699 | *FBXL14* | NA | 1 | |  | |  | |  |
| 145376 | *PPP1R36* | 1 | 1 | |  | |  | |  |
| 146050 | *ZSCAN29* | NA | 1 | |  | |  | |  |
| 146712 | *B3GNTL1* | 1 | NA | |  | |  | |  |
| 148014 | *TTC9B* | NA | 1 | |  | |  | |  |
| 148362 | *BROX* | 1 | 1 | |  | |  | |  |
| 148398 | *SAMD11* | 1 | 1 | |  | |  | |  |
| 148741 | *ANKRD35* | 1 | NA | |  | |  | |  |
| 148753 | *FAM163A* | 1 | 1 | |  | |  | |  |
| 149233 | *IL23R* | 1 | NA | |  | |  | |  |
| 150538 | *SATB2-AS1* | 1 | NA | |  | |  | |  |
| 152992 | *TRMT44* | 1 | 1 | |  | |  | |  |
| 153090 | *DAB2IP* | 1 | NA | |  | |  | |  |
| 153768 | *PRELID2* | NA | 1 | |  | |  | |  |
| 1592 | *CYP26A1* | 1 | 1 | |  | |  | |  |
| 1613 | *DAPK3* | 1 | 1 | |  | |  | |  |
| 161424 | *NOP9* | NA | 1 | |  | |  | |  |
| 162461 | *TMEM92* | NA | 1 | |  | |  | |  |
| 1639 | *DCTN1* | 1 | NA | |  | |  | |  |
| 168090 | *C6orf118* | 1 | NA | |  | |  | |  |
| 1838 | *DTNB* | 1 | NA | |  | |  | |  |
| 1850 | *DUSP8* | NA | 1 | |  | |  | |  |
| 1854 | *DUT* | NA | 1 | |  | |  | |  |
| 1890 | *TYMP* | 1 | 1 | |  | |  | |  |
| 199990 | *FAAP20* | 1 | NA | |  | |  | |  |
| 2000 | *ELF4* | NA | 1 | |  | |  | |  |
| 2003 | *ELK2AP* | 1 | NA | |  | |  | |  |
| 200424 | *TET3* | 1 | NA | |  | |  | |  |
| 201514 | *ZNF584* | 1 | 1 | |  | |  | |  |
| 2035 | *EPB41* | 1 | NA | |  | |  | |  |
| 2048 | *EPHB2* | 1 | NA | |  | |  | |  |
| 204851 | *HIPK1* | 1 | 1 | |  | |  | |  |
| 210 | *ALAD* | NA | 1 | |  | |  | |  |
| 2104 | *ESRRG* | 1 | NA | |  | |  | |  |
| 2120 | *ETV6* | NA | 1 | |  | |  | |  |
| 2130 | *EWSR1* | 1 | 1 | |  | |  | |  |
| 219670 | *ENKUR* | 1 | 1 | |  | |  | |  |
| 220064 | *ORAOV1* | NA | 1 | |  | |  | |  |
| 220359 | *TIGD3* | 1 | 1 | |  | |  | |  |
| 221060 | *C10orf111* | NA | 1 | |  | |  | |  |
| 221150 | *SKA3* | 1 | NA | |  | |  | |  |
| 221178 | *SPATA13* | 1 | NA | |  | |  | |  |
| 222643 | *UNC5CL* | 1 | 1 | |  | |  | |  |
| 225 | *ABCD2* | 1 | 1 | |  | |  | |  |
| 2253 | *FGF8* | 1 | NA | |  | |  | |  |
| 22801 | *ITGA11* | 1 | NA | |  | |  | |  |
| 22848 | *AAK1* | 1 | NA | |  | |  | |  |
| 22889 | *KHDC4* | 1 | 1 | |  | |  | |  |
| 22906 | *TRAK1* | 1 | 1 | |  | |  | |  |
| 22928 | *SEPHS2* | 1 | 1 | |  | |  | |  |
| 22948 | *CCT5* | 1 | NA | |  | |  | |  |
| 22950 | *SLC4A1AP* | 1 | 1 | |  | |  | |  |
| 22996 | *TTC39A* | 1 | NA | |  | |  | |  |
| 23001 | *WDFY3* | 1 | NA | |  | |  | |  |
| 23033 | *DOPEY1* | NA | 1 | |  | |  | |  |
| 23126 | *POGZ* | 1 | NA | |  | |  | |  |
| 23143 | *LRCH1* | 1 | NA | |  | |  | |  |
| 23179 | *RGL1* | 1 | NA | |  | |  | |  |
| 23191 | *CYFIP1* | NA | 1 | |  | |  | |  |
| 23208 | *SYT11* | 1 | NA | |  | |  | |  |
| 2326 | *FMO1* | 1 | NA | |  | |  | |  |
| 23266 | *ADGRL2* | 1 | NA | |  | |  | |  |
| 2327 | *FMO2* | 1 | NA | |  | |  | |  |
| 23294 | *ANKS1A* | 1 | NA | |  | |  | |  |
| 23303 | *KIF13B* | 1 | NA | |  | |  | |  |
| 23312 | *DMXL2* | 1 | NA | |  | |  | |  |
| 23314 | *SATB2* | NA | 1 | |  | |  | |  |
| 23329 | *TBC1D30* | NA | 1 | |  | |  | |  |
| 23352 | *UBR4* | NA | 1 | |  | |  | |  |
| 23353 | *SUN1* | 1 | NA | |  | |  | |  |
| 23378 | *RRP8* | NA | 1 | |  | |  | |  |
| 23406 | *COTL1* | 1 | NA | |  | |  | |  |
| 2342 | *FNTB* | NA | 1 | |  | |  | |  |
| 23432 | *GPR161* | 1 | 1 | |  | |  | |  |
| 23492 | *CBX7* | NA | 1 | |  | |  | |  |
| 23554 | *TSPAN12* | NA | 1 | |  | |  | |  |
| 23705 | *CADM1* | 1 | NA | |  | |  | |  |
| 238 | *ALK* | 1 | NA | |  | |  | |  |
| 24142 | *NAT6* | 1 | 1 | |  | |  | |  |
| 254394 | *MCM9* | 1 | 1 | |  | |  | |  |
| 2548 | *GAA* | 1 | NA | |  | |  | |  |
| 256356 | *GK5* | NA | 1 | |  | |  | |  |
| 25807 | *RHBDD3* | NA | 1 | |  | |  | |  |
| 259217 | *HSPA12A* | 1 | NA | |  | |  | |  |
| 25939 | *SAMHD1* | NA | 1 | |  | |  | |  |
| 25941 | *TPGS2* | 1 | NA | |  | |  | |  |
| 25956 | *SEC31B* | 1 | NA | |  | |  | |  |
| 26057 | *ANKRD17* | 1 | NA | |  | |  | |  |
| 26092 | *TOR1AIP1* | 1 | NA | |  | |  | |  |
| 26119 | *LDLRAP1* | 1 | NA | |  | |  | |  |
| 26136 | *TES* | 1 | NA | |  | |  | |  |
| 26207 | *PITPNC1* | 1 | NA | |  | |  | |  |
| 26520 | *TIMM9* | 1 | NA | |  | |  | |  |
| 26801 | *SNORD48* | NA | 1 | |  | |  | |  |
| 26828 | *RNU5F-1* | 1 | NA | |  | |  | |  |
| 26974 | *ZNF285* | NA | 1 | |  | |  | |  |
| 26985 | *AP3M1* | NA | 1 | |  | |  | |  |
| 26995 | *TRUB2* | NA | 1 | |  | |  | |  |
| 271 | *AMPD2* | 1 | 1 | |  | |  | |  |
| 27102 | *EIF2AK1* | 1 | NA | |  | |  | |  |
| 27229 | *TUBGCP4* | NA | 1 | |  | |  | |  |
| 27237 | *ARHGEF16* | 1 | NA | |  | |  | |  |
| 27243 | *CHMP2A* | NA | 1 | |  | |  | |  |
| 27255 | *CNTN6* | 1 | NA | |  | |  | |  |
| 27327 | *TNRC6A* | 1 | 1 | |  | |  | |  |
| 27346 | *TMEM97* | NA | 1 | |  | |  | |  |
| 2774 | *GNAL* | 1 | NA | |  | |  | |  |
| 2788 | *GNG7* | 1 | NA | |  | |  | |  |
| 283102 | *KRT8P41* | NA | 1 | |  | |  | |  |
| 283554 | *GPR137C* | NA | 1 | |  | |  | |  |
| 283856 | *LOC283856* | 1 | NA | |  | |  | |  |
| 284069 | *FAM171A2* | NA | 1 | |  | |  | |  |
| 284098 | *PIGW* | 1 | NA | |  | |  | |  |
| 284273 | *ZADH2* | 1 | NA | |  | |  | |  |
| 284352 | *PPP1R37* | NA | 1 | |  | |  | |  |
| 285116 | *AHCTF1P1* | 1 | NA | |  | |  | |  |
| 28514 | *DLL1* | 1 | NA | |  | |  | |  |
| 285590 | *SH3PXD2B* | 1 | NA | |  | |  | |  |
| 285593 | *LOC285593* | 1 | NA | |  | |  | |  |
| 2895 | *GRID2* | 1 | NA | |  | |  | |  |
| 28974 | *C19orf53* | 1 | NA | |  | |  | |  |
| 2900 | *GRIK4* | 1 | NA | |  | |  | |  |
| 2918 | *GRM8* | 1 | 1 | |  | |  | |  |
| 29993 | *PACSIN1* | NA | 1 | |  | |  | |  |
| 3084 | *NRG1* | 1 | NA | |  | |  | |  |
| 3092 | *HIP1* | 1 | NA | |  | |  | |  |
| 3106 | *HLA-B* | NA | 1 | |  | |  | |  |
| 3148 | *HMGB2* | NA | 1 | |  | |  | |  |
| 317751 | *MESTIT1* | NA | 1 | |  | |  | |  |
| 3185 | *HNRNPF* | 1 | 1 | |  | |  | |  |
| 3205 | *HOXA9* | 1 | NA | |  | |  | |  |
| 321 | *APBA2* | 1 | NA | |  | |  | |  |
| 330 | *BIRC3* | NA | 1 | |  | |  | |  |
| 334 | *APLP2* | NA | 1 | |  | |  | |  |
| 3361 | *HTR5A* | 1 | NA | |  | |  | |  |
| 337968 | *KRTAP6-3* | NA | 1 | |  | |  | |  |
| 338651 | *KRTAP5-AS1* | 1 | 1 | |  | |  | |  |
| 339 | *APOBEC1* | 1 | NA | |  | |  | |  |
| 339416 | *ANKRD45* | 1 | NA | |  | |  | |  |
| 342977 | *NANOS3* | 1 | NA | |  | |  | |  |
| 343099 | *CCDC18* | 1 | NA | |  | |  | |  |
| 347689 | *SOX2-OT* | 1 | NA | |  | |  | |  |
| 348262 | *MCRIP1* | NA | 1 | |  | |  | |  |
| 350383 | *GPR142* | NA | 1 | |  | |  | |  |
| 3611 | *ILK* | 1 | 1 | |  | |  | |  |
| 3678 | *ITGA5* | 1 | 1 | |  | |  | |  |
| 3707 | *ITPKB* | 1 | NA | |  | |  | |  |
| 3720 | *JARID2* | 1 | 1 | |  | |  | |  |
| 3748 | *KCNC3* | 1 | 1 | |  | |  | |  |
| 374928 | *ZNF773* | NA | 1 | |  | |  | |  |
| 375033 | *PEAR1* | 1 | NA | |  | |  | |  |
| 375196 | *LOC375196* | NA | 1 | |  | |  | |  |
| 375387 | *NRROS* | 1 | NA | |  | |  | |  |
| 376267 | *RAB15* | NA | 1 | |  | |  | |  |
| 376497 | *SLC27A1* | 1 | NA | |  | |  | |  |
| 3784 | *KCNQ1* | 1 | NA | |  | |  | |  |
| 38 | *ACAT1* | 1 | 1 | |  | |  | |  |
| 388394 | *RPRML* | NA | 1 | |  | |  | |  |
| 389337 | *ARHGEF37* | NA | 1 | |  | |  | |  |
| 390010 | *NKX1-2* | 1 | NA | |  | |  | |  |
| 3930 | *LBR* | NA | 1 | |  | |  | |  |
| 3932 | *LCK* | 1 | NA | |  | |  | |  |
| 399665 | *FAM102A* | 1 | 1 | |  | |  | |  |
| 4004 | *LMO1* | 1 | 1 | |  | |  | |  |
| 401145 | *CCSER1* | 1 | 1 | |  | |  | |  |
| 401898 | *ZNF833P* | 1 | NA | |  | |  | |  |
| 404093 | *CUEDC1* | NA | 1 | |  | |  | |  |
| 407015 | *MIR26A1* | NA | 1 | |  | |  | |  |
| 4148 | *MATN3* | NA | 1 | |  | |  | |  |
| 4176 | *MCM7* | 1 | NA | |  | |  | |  |
| 4223 | *MEOX2* | 1 | NA | |  | |  | |  |
| 4232 | *MEST* | 1 | 1 | |  | |  | |  |
| 4240 | *MFGE8* | 1 | NA | |  | |  | |  |
| 4297 | *KMT2A* | NA | 1 | |  | |  | |  |
| 4329 | *ALDH6A1* | NA | 1 | |  | |  | |  |
| 440119 | *FZD10-DT* | 1 | NA | |  | |  | |  |
| 4430 | *MYO1B* | 1 | NA | |  | |  | |  |
| 475 | *ATOX1* | NA | 1 | |  | |  | |  |
| 4750 | *NEK1* | 1 | 1 | |  | |  | |  |
| 4782 | *NFIC* | 1 | 1 | |  | |  | |  |
| 4901 | *NRL* | 1 | NA | |  | |  | |  |
| 4919 | *ROR1* | NA | 1 | |  | |  | |  |
| 494326 | *MIR377* | NA | 1 | |  | |  | |  |
| 49854 | *ZBTB21* | NA | 1 | |  | |  | |  |
| 5045 | *FURIN* | 1 | NA | |  | |  | |  |
| 50804 | *MYEF2* | 1 | 1 | |  | |  | |  |
| 5081 | *PAX7* | NA | 1 | |  | |  | |  |
| 50836 | *TAS2R8* | NA | 1 | |  | |  | |  |
| 50854 | *C6orf48* | NA | 1 | |  | |  | |  |
| 50940 | *PDE11A* | 1 | 1 | |  | |  | |  |
| 50999 | *TMED5* | NA | 1 | |  | |  | |  |
| 51019 | *WASHC3* | NA | 1 | |  | |  | |  |
| 51069 | *MRPL2* | 1 | 1 | |  | |  | |  |
| 51086 | *TNNI3K* | 1 | NA | |  | |  | |  |
| 51117 | *COQ4* | 1 | NA | |  | |  | |  |
| 51160 | *VPS28* | NA | 1 | |  | |  | |  |
| 51161 | *C3orf18* | NA | 1 | |  | |  | |  |
| 51188 | *SS18L2* | NA | 1 | |  | |  | |  |
| 51191 | *HERC5* | 1 | 1 | |  | |  | |  |
| 51334 | *PRR16* | 1 | NA | |  | |  | |  |
| 51373 | *MRPS17* | NA | 1 | |  | |  | |  |
| 51409 | *HEMK1* | 1 | NA | |  | |  | |  |
| 51430 | *SUCO* | NA | 1 | |  | |  | |  |
| 5158 | *PDE6B* | 1 | NA | |  | |  | |  |
| 5159 | *PDGFRB* | NA | 1 | |  | |  | |  |
| 51593 | *SRRT* | NA | 1 | |  | |  | |  |
| 51701 | *NLK* | 1 | NA | |  | |  | |  |
| 5217 | *PFN2* | 1 | 1 | |  | |  | |  |
| 523 | *ATP6V1A* | 1 | 1 | |  | |  | |  |
| 5295 | *PIK3R1* | 1 | 1 | |  | |  | |  |
| 53343 | *NUDT9* | NA | 1 | |  | |  | |  |
| 5356 | *PLRG1* | NA | 1 | |  | |  | |  |
| 5359 | *PLSCR1* | NA | 1 | |  | |  | |  |
| 5365 | *PLXNB3* | 1 | NA | |  | |  | |  |
| 54434 | *SSH1* | 1 | 1 | |  | |  | |  |
| 54487 | *DGCR8* | 1 | 1 | |  | |  | |  |
| 54502 | *RBM47* | 1 | NA | |  | |  | |  |
| 54503 | *ZDHHC13* | NA | 1 | |  | |  | |  |
| 54758 | *KLHDC4* | 1 | NA | |  | |  | |  |
| 54795 | *TRPM4* | 1 | NA | |  | |  | |  |
| 54811 | *ZNF562* | NA | 1 | |  | |  | |  |
| 54897 | *CASZ1* | 1 | NA | |  | |  | |  |
| 54940 | *OCIAD1* | NA | 1 | |  | |  | |  |
| 55033 | *FKBP14* | NA | 1 | |  | |  | |  |
| 55036 | *CCDC40* | 1 | NA | |  | |  | |  |
| 55062 | *WIPI1* | 1 | NA | |  | |  | |  |
| 55068 | *ENOX1* | 1 | NA | |  | |  | |  |
| 55088 | *CCDC186* | NA | 1 | |  | |  | |  |
| 55114 | *ARHGAP17* | 1 | 1 | |  | |  | |  |
| 55127 | *HEATR1* | NA | 1 | |  | |  | |  |
| 55159 | *RFWD3* | NA | 1 | |  | |  | |  |
| 55160 | *ARHGEF10L* | 1 | NA | |  | |  | |  |
| 55276 | *PGM2* | 1 | NA | |  | |  | |  |
| 55283 | *MCOLN3* | NA | 1 | |  | |  | |  |
| 553103 | *MIR3936HG* | 1 | 1 | |  | |  | |  |
| 55315 | *SLC29A3* | NA | 1 | |  | |  | |  |
| 55336 | *FBXL8* | 1 | 1 | |  | |  | |  |
| 55361 | *PI4K2A* | 1 | 1 | |  | |  | |  |
| 55532 | *SLC30A10* | 1 | NA | |  | |  | |  |
| 55611 | *OTUB1* | NA | 1 | |  | |  | |  |
| 5562 | *PRKAA1* | NA | 1 | |  | |  | |  |
| 55635 | *DEPDC1* | 1 | 1 | |  | |  | |  |
| 5575 | *PRKAR1B* | 1 | NA | |  | |  | |  |
| 55758 | *RCOR3* | 1 | NA | |  | |  | |  |
| 55790 | *CSGALNACT1* | NA | 1 | |  | |  | |  |
| 55819 | *RNF130* | 1 | 1 | |  | |  | |  |
| 55854 | *ZC3H15* | NA | 1 | |  | |  | |  |
| 55902 | *ACSS2* | 1 | 1 | |  | |  | |  |
| 55924 | *FAM212B* | 1 | NA | |  | |  | |  |
| 56005 | *MYDGF* | 1 | 1 | |  | |  | |  |
| 56134 | *PCDHAC2* | 1 | 1 | |  | |  | |  |
| 56135 | *PCDHAC1* | 1 | NA | |  | |  | |  |
| 56136 | *PCDHA13* | 1 | NA | |  | |  | |  |
| 56137 | *PCDHA12* | 1 | NA | |  | |  | |  |
| 56138 | *PCDHA11* | 1 | NA | |  | |  | |  |
| 56139 | *PCDHA10* | 1 | NA | |  | |  | |  |
| 56140 | *PCDHA8* | 1 | NA | |  | |  | |  |
| 56141 | *PCDHA7* | 1 | NA | |  | |  | |  |
| 56142 | *PCDHA6* | 1 | NA | |  | |  | |  |
| 56143 | *PCDHA5* | 1 | NA | |  | |  | |  |
| 56144 | *PCDHA4* | 1 | NA | |  | |  | |  |
| 56145 | *PCDHA3* | 1 | NA | |  | |  | |  |
| 56146 | *PCDHA2* | 1 | NA | |  | |  | |  |
| 56147 | *PCDHA1* | 1 | NA | |  | |  | |  |
| 56474 | *CTPS2* | NA | 1 | |  | |  | |  |
| 56704 | *JPH1* | NA | 1 | |  | |  | |  |
| 56853 | *CELF4* | 1 | NA | |  | |  | |  |
| 5687 | *PSMA6* | NA | 1 | |  | |  | |  |
| 56886 | *UGGT1* | 1 | 1 | |  | |  | |  |
| 56937 | *PMEPA1* | 1 | NA | |  | |  | |  |
| 56950 | *SMYD2* | 1 | NA | |  | |  | |  |
| 57030 | *SLC17A7* | 1 | 1 | |  | |  | |  |
| 57132 | *CHMP1B* | 1 | 1 | |  | |  | |  |
| 57150 | *SMIM8* | 1 | NA | |  | |  | |  |
| 57209 | *ZNF248* | 1 | NA | |  | |  | |  |
| 57337 | *SENP7* | NA | 1 | |  | |  | |  |
| 57348 | *TTYH1* | 1 | NA | |  | |  | |  |
| 57379 | *AICDA* | 1 | NA | |  | |  | |  |
| 574481 | *MIR521-2* | NA | 1 | |  | |  | |  |
| 57474 | *ZNF490* | 1 | NA | |  | |  | |  |
| 57513 | *CASKIN2* | 1 | NA | |  | |  | |  |
| 57536 | *KIAA1328* | NA | 1 | |  | |  | |  |
| 57544 | *TXNDC16* | NA | 1 | |  | |  | |  |
| 57553 | *MICAL3* | 1 | NA | |  | |  | |  |
| 57556 | *SEMA6A* | NA | 1 | |  | |  | |  |
| 57569 | *ARHGAP20* | 1 | 1 | |  | |  | |  |
| 57575 | *PCDH10* | NA | 1 | |  | |  | |  |
| 57582 | *KCNT1* | 1 | NA | |  | |  | |  |
| 57620 | *STIM2* | 1 | NA | |  | |  | |  |
| 57654 | *UVSSA* | 1 | NA | |  | |  | |  |
| 57666 | *FBRSL1* | 1 | NA | |  | |  | |  |
| 57693 | *ZNF317* | 1 | NA | |  | |  | |  |
| 57758 | *SCUBE2* | NA | 1 | |  | |  | |  |
| 5789 | *PTPRD* | 1 | NA | |  | |  | |  |
| 5793 | *PTPRG* | 1 | NA | |  | |  | |  |
| 5816 | *PVALB* | 1 | 1 | |  | |  | |  |
| 583 | *BBS2* | NA | 1 | |  | |  | |  |
| 5832 | *ALDH18A1* | 1 | 1 | |  | |  | |  |
| 5977 | *DPF2* | NA | 1 | |  | |  | |  |
| 5990 | *RFX2* | 1 | NA | |  | |  | |  |
| 5993 | *RFX5* | NA | 1 | |  | |  | |  |
| 6095 | *RORA* | 1 | NA | |  | |  | |  |
| 619343 | *NA* | 1 | NA | |  | |  | |  |
| 6262 | *RYR2* | 1 | NA | |  | |  | |  |
| 63027 | *SLC22A23* | NA | 1 | |  | |  | |  |
| 6329 | *SCN4A* | NA | 1 | |  | |  | |  |
| 639 | *PRDM1* | NA | 1 | |  | |  | |  |
| 63976 | *PRDM16* | 1 | NA | |  | |  | |  |
| 642366 | *LOC642366* | 1 | NA | |  | |  | |  |
| 64328 | *XPO4* | 1 | 1 | |  | |  | |  |
| 64377 | *CHST8* | 1 | NA | |  | |  | |  |
| 643803 | *KRTAP24-1* | 1 | NA | |  | |  | |  |
| 64478 | *CSMD1* | 1 | NA | |  | |  | |  |
| 646903 | *LOC646903* | NA | 1 | |  | |  | |  |
| 64746 | *ACBD3* | NA | 1 | |  | |  | |  |
| 64786 | *TBC1D15* | NA | 1 | |  | |  | |  |
| 64853 | *AIDA* | NA | 1 | |  | |  | |  |
| 64881 | *PCDH20* | 1 | NA | |  | |  | |  |
| 64919 | *BCL11B* | NA | 1 | |  | |  | |  |
| 6497 | *SKI* | 1 | NA | |  | |  | |  |
| 65012 | *SLC26A10* | NA | 1 | |  | |  | |  |
| 65108 | *MARCKSL1* | NA | 1 | |  | |  | |  |
| 6514 | *SLC2A2* | NA | 1 | |  | |  | |  |
| 652276 | *LOC652276* | NA | 1 | |  | |  | |  |
| 6584 | *SLC22A5* | NA | 1 | |  | |  | |  |
| 6585 | *SLIT1* | NA | 1 | |  | |  | |  |
| 6588 | *SLN* | NA | 1 | |  | |  | |  |
| 65985 | *AACS* | 1 | 1 | |  | |  | |  |
| 65996 | *CENPBD1P1* | 1 | NA | |  | |  | |  |
| 66005 | *CHID1* | NA | 1 | |  | |  | |  |
| 6604 | *SMARCD3* | 1 | NA | |  | |  | |  |
| 6631 | *SNRPC* | 1 | 1 | |  | |  | |  |
| 6648 | *SOD2* | 1 | NA | |  | |  | |  |
| 6687 | *SPG7* | 1 | 1 | |  | |  | |  |
| 6788 | *STK3* | 1 | NA | |  | |  | |  |
| 6865 | *TACR2* | 1 | NA | |  | |  | |  |
| 6867 | *TACC1* | 1 | 1 | |  | |  | |  |
| 6892 | *TAPBP* | NA | 1 | |  | |  | |  |
| 6904 | *TBCD* | 1 | 1 | |  | |  | |  |
| 693220 | *MIR635* | NA | 1 | |  | |  | |  |
| 699 | *BUB1* | 1 | NA | |  | |  | |  |
| 7003 | *TEAD1* | 1 | NA | |  | |  | |  |
| 7020 | *TFAP2A* | 1 | NA | |  | |  | |  |
| 7025 | *NR2F1* | 1 | 1 | |  | |  | |  |
| 7068 | *THRB* | 1 | NA | |  | |  | |  |
| 7077 | *TIMP2* | 1 | NA | |  | |  | |  |
| 715 | *C1R* | 1 | NA | |  | |  | |  |
| 7164 | *TPD52L1* | 1 | 1 | |  | |  | |  |
| 7226 | *TRPM2* | 1 | 1 | |  | |  | |  |
| 7262 | *PHLDA2* | NA | 1 | |  | |  | |  |
| 7277 | *TUBA4A* | 1 | NA | |  | |  | |  |
| 728192 | *LINC00460* | 1 | NA | |  | |  | |  |
| 728912 | *NA* | 1 | NA | |  | |  | |  |
| 728932 | *NA* | 1 | NA | |  | |  | |  |
| 728939 | *NA* | 1 | NA | |  | |  | |  |
| 7323 | *UBE2D3* | 1 | NA | |  | |  | |  |
| 7342 | *UBP1* | 1 | 1 | |  | |  | |  |
| 7444 | *VRK2* | 1 | NA | |  | |  | |  |
| 747 | *DAGLA* | NA | 1 | |  | |  | |  |
| 7520 | *XRCC5* | 1 | NA | |  | |  | |  |
| 768 | *CA9* | 1 | NA | |  | |  | |  |
| 773 | *CACNA1A* | 1 | NA | |  | |  | |  |
| 7771 | *ZNF112* | NA | 1 | |  | |  | |  |
| 78988 | *MRPL57* | NA | 1 | |  | |  | |  |
| 79018 | *GID4* | 1 | NA | |  | |  | |  |
| 79087 | *ALG12* | NA | 1 | |  | |  | |  |
| 7915 | *ALDH5A1* | 1 | NA | |  | |  | |  |
| 79174 | *CRELD2* | 1 | 1 | |  | |  | |  |
| 79696 | *ZC2HC1C* | NA | 1 | |  | |  | |  |
| 79703 | *C11orf80* | 1 | NA | |  | |  | |  |
| 79745 | *CLIP4* | 1 | 1 | |  | |  | |  |
| 79791 | *FBXO31* | 1 | NA | |  | |  | |  |
| 79812 | *MMRN2* | 1 | NA | |  | |  | |  |
| 79832 | *QSER1* | 1 | NA | |  | |  | |  |
| 79890 | *RIN3* | NA | 1 | |  | |  | |  |
| 79896 | *THNSL1* | NA | 1 | |  | |  | |  |
| 79913 | *ACTR5* | 1 | NA | |  | |  | |  |
| 79918 | *SETD6* | 1 | NA | |  | |  | |  |
| 79919 | *C2orf54* | 1 | NA | |  | |  | |  |
| 79960 | *JADE1* | 1 | NA | |  | |  | |  |
| 79977 | *GRHL2* | 1 | NA | |  | |  | |  |
| 80005 | *DOCK5* | 1 | NA | |  | |  | |  |
| 8001 | *GLRA3* | NA | 1 | |  | |  | |  |
| 80086 | *TUBA4B* | NA | 1 | |  | |  | |  |
| 80125 | *CCDC33* | 1 | NA | |  | |  | |  |
| 80176 | *SPSB1* | 1 | NA | |  | |  | |  |
| 8021 | *NUP214* | 1 | NA | |  | |  | |  |
| 80218 | *NAA50* | NA | 1 | |  | |  | |  |
| 8022 | *LHX3* | 1 | NA | |  | |  | |  |
| 80312 | *TET1* | 1 | NA | |  | |  | |  |
| 80728 | *ARHGAP39* | NA | 1 | |  | |  | |  |
| 80760 | *ITIH5* | 1 | 1 | |  | |  | |  |
| 81029 | *WNT5B* | 1 | NA | |  | |  | |  |
| 81532 | *MOB2* | 1 | NA | |  | |  | |  |
| 8174 | *MADCAM1* | 1 | NA | |  | |  | |  |
| 8310 | *ACOX3* | 1 | 1 | |  | |  | |  |
| 83538 | *TTC25* | NA | 1 | |  | |  | |  |
| 83659 | *TEKT1* | NA | 1 | |  | |  | |  |
| 8369 | *HIST1H4G* | 1 | 1 | |  | |  | |  |
| 8372 | *HYAL3* | 1 | 1 | |  | |  | |  |
| 83943 | *IMMP2L* | 1 | NA | |  | |  | |  |
| 84033 | *OBSCN* | 1 | NA | |  | |  | |  |
| 84056 | *KATNAL1* | 1 | NA | |  | |  | |  |
| 84067 | *FAM160A2* | NA | 1 | |  | |  | |  |
| 84068 | *SLC10A7* | NA | 1 | |  | |  | |  |
| 84080 | *ENKD1* | 1 | NA | |  | |  | |  |
| 84126 | *ATRIP* | 1 | 1 | |  | |  | |  |
| 84166 | *NLRC5* | 1 | NA | |  | |  | |  |
| 84236 | *RHBDD1* | 1 | NA | |  | |  | |  |
| 84264 | *HAGHL* | 1 | NA | |  | |  | |  |
| 84272 | *YIPF4* | NA | 1 | |  | |  | |  |
| 84284 | *NTPCR* | NA | 1 | |  | |  | |  |
| 84302 | *TMEM246* | NA | 1 | |  | |  | |  |
| 84316 | *NAA38* | NA | 1 | |  | |  | |  |
| 84435 | *ADGRA1* | NA | 1 | |  | |  | |  |
| 8460 | *TPST1* | NA | 1 | |  | |  | |  |
| 8470 | *SORBS2* | 1 | 1 | |  | |  | |  |
| 84725 | *PLEKHA8* | 1 | 1 | |  | |  | |  |
| 84856 | *LINC00839* | 1 | 1 | |  | |  | |  |
| 84858 | *ZNF503* | NA | 1 | |  | |  | |  |
| 84929 | *FIBCD1* | 1 | NA | |  | |  | |  |
| 8495 | *PPFIBP2* | 1 | NA | |  | |  | |  |
| 85001 | *MGC16275* | 1 | NA | |  | |  | |  |
| 8532 | *CPZ* | 1 | NA | |  | |  | |  |
| 8536 | *CAMK1* | NA | 1 | |  | |  | |  |
| 85403 | *EAF1* | NA | 1 | |  | |  | |  |
| 857 | *CAV1* | 1 | 1 | |  | |  | |  |
| 8601 | *RGS20* | 1 | NA | |  | |  | |  |
| 864 | *RUNX3* | 1 | NA | |  | |  | |  |
| 8661 | *EIF3A* | 1 | NA | |  | |  | |  |
| 8664 | *EIF3D* | NA | 1 | |  | |  | |  |
| 8677 | *STX10* | NA | 1 | |  | |  | |  |
| 8717 | *TRADD* | NA | 1 | |  | |  | |  |
| 8736 | *MYOM1* | 1 | NA | |  | |  | |  |
| 8775 | *NAPA* | NA | 1 | |  | |  | |  |
| 8835 | *SOCS2* | 1 | 1 | |  | |  | |  |
| 8927 | *BSN* | NA | 1 | |  | |  | |  |
| 894 | *CCND2* | NA | 1 | |  | |  | |  |
| 8943 | *AP3D1* | NA | 1 | |  | |  | |  |
| 89953 | *KLC4* | 1 | 1 | |  | |  | |  |
| 90025 | *UBE3D* | 1 | 1 | |  | |  | |  |
| 902 | *CCNH* | NA | 1 | |  | |  | |  |
| 90427 | *BMF* | 1 | NA | |  | |  | |  |
| 90525 | *SHF* | 1 | NA | |  | |  | |  |
| 9117 | *SEC22C* | 1 | NA | |  | |  | |  |
| 91408 | *BTF3L4* | 1 | NA | |  | |  | |  |
| 9141 | *PDCD5* | NA | 1 | |  | |  | |  |
| 91452 | *ACBD5* | 1 | 1 | |  | |  | |  |
| 91603 | *ZNF830* | NA | 1 | |  | |  | |  |
| 91748 | *ELMSAN1* | 1 | 1 | |  | |  | |  |
| 91750 | *LIN52* | NA | 1 | |  | |  | |  |
| 91775 | *NXPE3* | 1 | NA | |  | |  | |  |
| 92345 | *NAF1* | 1 | 1 | |  | |  | |  |
| 9249 | *DHRS3* | 1 | NA | |  | |  | |  |
| 92521 | *SPECC1* | 1 | 1 | |  | |  | |  |
| 928 | *CD9* | 1 | NA | |  | |  | |  |
| 9364 | *RAB28* | NA | 1 | |  | |  | |  |
| 93974 | *ATP5IF1* | 1 | 1 | |  | |  | |  |
| 94015 | *TTYH2* | NA | 1 | |  | |  | |  |
| 9423 | *NTN1* | 1 | NA | |  | |  | |  |
| 9440 | *MED17* | NA | 1 | |  | |  | |  |
| 9443 | *MED7* | NA | 1 | |  | |  | |  |
| 9538 | *EI24* | 1 | NA | |  | |  | |  |
| 9570 | *GOSR2* | 1 | NA | |  | |  | |  |
| 9578 | *CDC42BPB* | 1 | NA | |  | |  | |  |
| 9592 | *IER2* | 1 | 1 | |  | |  | |  |
| 960 | *CD44* | 1 | NA | |  | |  | |  |
| 9644 | *SH3PXD2A* | 1 | NA | |  | |  | |  |
| 9692 | *KIAA0391* | 1 | NA | |  | |  | |  |
| 9703 | *KIAA0100* | 1 | 1 | |  | |  | |  |
| 9718 | *ECE2* | 1 | NA | |  | |  | |  |
| 9747 | *TCAF1* | 1 | 1 | |  | |  | |  |
| 9752 | *PCDHA9* | 1 | NA | |  | |  | |  |
| 9759 | *HDAC4* | 1 | 1 | |  | |  | |  |
| 9761 | *MLEC* | 1 | NA | |  | |  | |  |
| 9913 | *SUPT7L* | 1 | 1 | |  | |  | |  |
| 9924 | *PAN2* | 1 | NA | |  | |  | |  |
| 9941 | *EXOG* | 1 | 1 | |  | |  | |  |
| 9986 | *RCE1* | NA | 1 | |  | |  | |  |

**Supplemental Table 4. Overlap of MD related DMPs/DMRs and VMPs/VMRs with PGC GWAS loci.**

| **Chromosome** | **DNAm.start** | **DNAm.end** | | **PGC.start** | **PGC.end** | |  |
| --- | --- | --- | --- | --- | --- | --- | --- |
| **DMPs/DMRs**  chr6 | 29521506 | 29521506 | | 27738000 | 32848000 | |  |
| chr6 | 30031455 | 30031455 | | 27738000 | 32848000 | |  |
| chr6 | 30519905 | 30521619 | | 27738000 | 32848000 | |  |
| chr6 | 31828260 | 31830030 | | 27738000 | 32848000 | |  |
| chr6 | 32797253 | 32798887 | | 27738000 | 32848000 | |  |
| chr10 | 106401479 | 106401479 | | 106397000 | 106904000 | |  |
| **VMPs/VMRs**  chr2 | 58265673 | 58265673 | 57765000 | | | 58485000 | |
| chr5 | 164658718 | 164658718 | 164440000 | | | 164789000 | |
| chr6 | 28706472 | 28706472 | 27738000 | | | 32848000 | |
| chr6 | 28890673 | 28890673 | 27738000 | | | 32848000 | |
| chr6 | 31324972 | 31324972 | 27738000 | | | 32848000 | |
| chr6 | 31707922 | 31707922 | 27738000 | | | 32848000 | |
| chr6 | 31802397 | 31802397 | 27738000 | | | 32848000 | |
| chr6 | 32049516 | 32049825 | 27738000 | | | 32848000 | |
| chr6 | 32055738 | 32055738 | 27738000 | | | 32848000 | |
| chr6 | 32789916 | 32789916 | 27738000 | | | 32848000 | |
| chr6 | 32818212 | 32818212 | 27738000 | | | 32848000 | |
| chr6 | 32847830 | 32847830 | 27738000 | | | 32848000 | |

**Supplemental References**

1. American Psychiatric Association. *Diagnostic and Statistical Manual of Mental Disorders, 5th Edition*. (2013).

2. Kendler, K. S., Neale, M. C., Kessler, R. C., Heath, A. C. & Eaves, L. J. A population-based twin study of major depression in women. The impact of varying definitions of illness. *Arch Gen Psychiatry* **49**, 257–266 (1992).

3. Meier, A. The research diagnostic criteria: historical background, development, validity, and reliability. *Can J Psychiatry* **24**, 167–178 (1979).

4. Endicott, J. & Spitzer, R. L. Use of the Research Diagnostic Criteria and the Schedule for Affective Disorders and Schizophrenia to study affective disorders. *Am J Psychiatry* **136**, 52–56 (1979).

5. Spitzer, R. L., Endicott, J. & Robins, E. Research diagnostic criteria. *Psychopharmacol Bull* **11**, 22–25 (1975).

6. Spitzer, R. L., Endicott, J. & Robins, E. Research diagnostic criteria: rationale and reliability. *Arch Gen Psychiatry* **35**, 773–782 (1978).

7. Kasriel, J. & Eaves, L. The zygosity of twins: further evidence on the agreement between diagnosis by blood groups and written questionnaires. *J Biosoc Sci* **8**, 263–266 (1976).

8. Jackson, R. W., Snieder, H., Davis, H. & Treiber, F. A. Determination of twin zygosity: a comparison of DNA with various questionnaire indices. *Twin Res* **4**, 12–18 (2001).

9. Joehanes, R. *et al.* Epigenetic Signatures of Cigarette Smoking. *Circ Cardiovasc Genet* **9**, 436–447 (2016).
